# Electrically Tunable Metamaterials Based on Multimaterial Nanowires Incorporating Transparent Conductive Oxides

**DOI:** 10.1038/s41598-017-09523-4

**Published:** 2017-08-30

**Authors:** Mohammad Mahdi Salary, Hossein Mosallaei

**Affiliations:** Metamaterials Laboratory, Northeastern University, Electrical and Computer Engineering Department, Boston, MA 02115 USA

## Abstract

We present novel design approaches for metasurfaces and metamaterials with electrical tunability offering real-time manipulation of light and serving as multifunctional devices in near-infrared frequency regime (at the specific wavelength of 1.55 *μ*m). For this purpose, we integrate indium-tin-oxide (ITO) as a tunable electro-optical material into multimaterial nanowires with metal-oxide-semiconductor and metal-insulator-metal configurations. In particular, an active metasurface operating in the transmission mode is designed which allows for modulation of the transmitted light phase over 280 degrees. This large phase modulation is afforded in the cost of low transmission efficiency. We demonstrate the use of such active metasurfaces for tunable bending and focusing in free-space. Moreover, we investigate the implementation of this material in deeply subwavelength multimaterial nanowires, which can yield strong variations in the effective refractive index by the virtue of internal homogenization enabling tunability of the performance in gradient refractive index metamaterials. In the theoretical modeling of these structures, we adopt a hierarchical multiscale approach by linking drift-diffusion transport model with the electromagnetic model which rigorously characterizes the electro-optical effects.

## Introduction

Light waves and fields can be manipulated through different devices and systems. The emergence of gradient metamaterials has provided more flexibility in the light manipulation. In particular, gradient metamaterials can be classified into two groups: gradient metasurfaces and gradient refractive index (GRIN) metamaterials. Gradient metasurfaces are two-dimensional arrays of subwavelength elements which can desirably tailor the phase or amplitude of the reflected or transmitted light based on localized resonant features of the building blocks^1–3^. On the other hand, GRIN metamaterials are two- or three- dimensional arrays of deeply subwavelength unit-cells which realize a gradient refractive index profile thus enabling bending the light waves in peculiar ways^4–6^. The extensive research on gradient metamaterials has led to a variety of flat and bulk optical components for different applications including focusing lenses^7, 8^, beam steering^9–11^ and holographic devices^12, 13^.

One of the major limitations of the traditional metamaterial designs is that their optical response is fixed once fabricated, tying them to a specific application and operating wavelength for which they are designed. In order to overcome this limitation, an immense effort has been put on the post-fabrication control of these structures through various mechanisms including mechanical reconfiguration^14, 15^, utilizing phase change materials (PCMs)^16, 17^, and using electro-optical materials such as liquid crystals^18, 19^, low-dimensional graphene^20, 21^ and transparent conducting oxides^22, 23^. Such mechanisms can allow for tuning the performance of optical devices, serving multiple functionalities, as well as multispectral and broadband operation. Among all these mechanisms, using electro-optical materials offer shorter latencies and faster control mechanism as well as larger tuning range, simply by applying an external bias. Different groups of electro-optical materials have different operating principles and operation frequency regimes.

Liquid crystals are ordered liquids, in which the direction of optical axis and the refractive index can be changed as functions of external fields. They have been used successfully in photonic crystals^18^ and metasurfaces^19^ for addressing tunability. The refractive index change has proved to be enough to create bandgaps and tunable defects in photonic crystals^18^. Moreover, recently it has been shown experimentally that amplitude modulations of up to 75% and a phase change of up to approximately *π* can be achieved^19^ by applying an AC voltage to the liquid crystal. However, liquid crystals have been mostly integrated into the proposed designs as a substrate or superstrate, posing practical issues for realizing graded patterns which limits their application. Moreover, the thickness of these materials requires to be relatively thick limiting their application for ultra-thin metasurfaces.

Low-dimensional graphene has also received significant attention in recent years as an electro-optical material due to its unique features such as broadband operation, extreme thinness, compatibility with silicon technology and large scale fabrication capability. The surface conductivity of a graphene sheet can be tuned by applying a gate voltage. This effect has been exploited to create flat tunable gradient index metamaterials for manipulation of surface plasmon polariton (SPP) waves in the mid-infrared and far-infrared frequency regimes^21^. Furthermore, the integration of graphene into metasurfaces has allowed for spectrally tunable absorption^20^ and modulation of reflection phase at a single frequency which comes at the cost of low amplitude efficiency^24^.

Another emerging group of electro-optical materials is transparent conducting oxides (TCOs) such as indium tin oxide (ITO) and indium zinc oxide (IZO). These materials are degenerately doped semiconductors that are widely used as transparent electrodes for optoelectronic applications^25^. In the account of its compatibility with mature semiconductor fabrication technologies and unique carrier-induced phase changes, ITO is an ideal candidate to add tunability to the optical components in the near-infrared (NIR) regime. It has been shown that applying an external bias across an ITO layer can yield strong changes in the complex refractive index of an ultrathin charge accumulation layer at the interface of ITO with an insulating material^22^. This effect has been observed for other highly doped semiconductors like silicon as well^26^. However, the unique property of ITO is that the real part of the permittivity can change its sign from positive to negative in the NIR spectrum by applying an increasing electrical bias, leading to substantial changes in the light propagation through coupling to epsilon near-zero (ENZ) region^22, 23^. Following the pioneering work by Atwater’s group^22^, ITO material has been extensively investigated for modulation of the amplitude and phase of the reflected light using active metasurface platforms^23, 27–32^.

**Figure 1:**
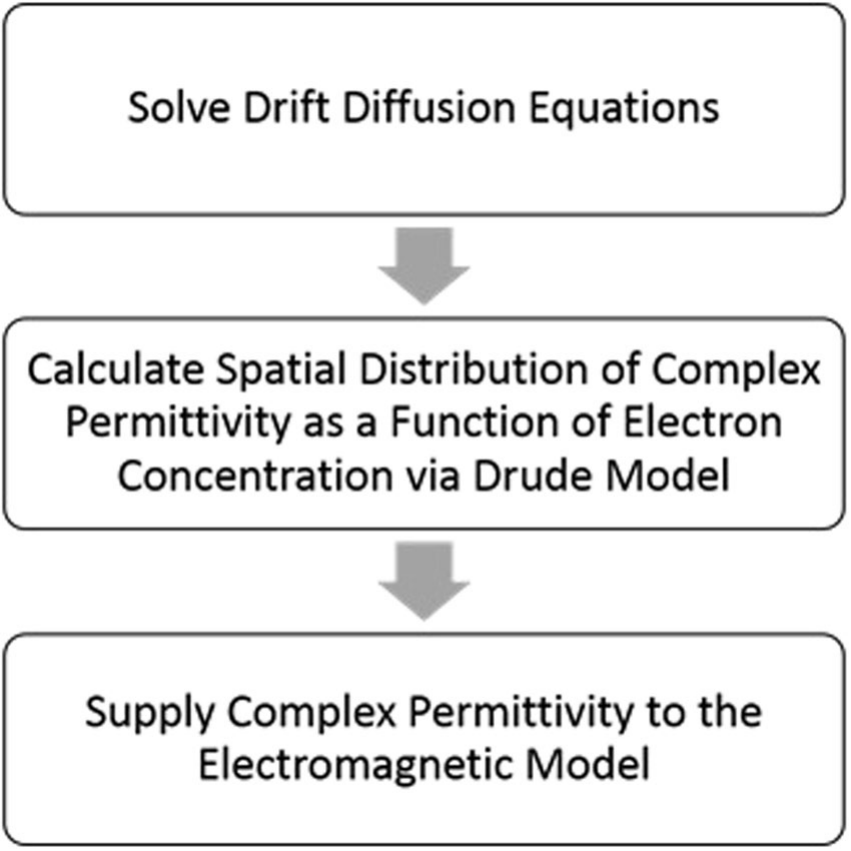
The hierarchical multiscale approach for modeling the electro-optical phenomena by linking drift-diffusion transport model and the electromagnetic model.

In this work, we introduce novel design approaches for ITO-based metamaterial platforms in the NIR regime to achieve multifunctionality and tunable performance in real-time. Specifically, we focus on the telecommunication wavelength of 1.55 *μ*m due to its importance. This is achieved by incorporating ITO into multimaterial nanowires in different configurations of metal-insulator-metal (MIM) and Metal-Oxide-Semiconductor (MOS). Such multimaterial nanowires are in the reach for fabrication with a great resolution using the current state of fabrication technology offering a variety of material compositions^33–36^ and vertical^37, 38^ or lateral^39, 40^ growth on a variety of substrates. MOS nanowires have been commonly used as preferable structures for MOS field-effect transistors (MOSFETs) due to possessing improved sub-threshold swings thanks to the curvature of the element^37–40^. A tunable metasurface capable of modulating the transmitted light phase over 280 degrees is designed. Despite the low amplitude efficiency of the proposed metasurface, the large afforded phase modulation can be used to realize a variety of functionalities on-demand through a graded-biasing pattern. Tunable bending and focusing in free-space are demonstrated as typical examples. In addition, we focus on tuning the effective refractive index of deeply subwavelength nanowires (with radii much smaller than the operating wavelength) through biasing multimaterial configurations incorporating ITO. A strong variation in the refractive index is achieved by the virtue of internal homogenization which can add tunability and multifunctionality to gradient index metamaterials. In order to rigorously characterize the electro-optical phenomena, we adopt a hierarchical multiscale modeling paradigm which links the drift-diffusion transport model to the electromagnetic model by calculating the spatial distribution of complex-valued permittivity of semiconductor materials as a function of electron concentration via Drude model and subsequently supplying it to the electromagnetic model. The overall procedure is shown in Fig. 1. For solving drift-diffusion equations, we utilize the open-source Cogenda device simulator^41^ which is a finite-element based drift-diffusion solver. An aggregate transition-matrix (T-matrix) approach^42^ is then used for electromagnetic modeling of substrate-supported arrays of multilayer inhomogeneous nanowires^43–45^. Owing to its superior efficiency compared to brute-force computational methods, the T-matrix model facilitates the performance characterization of electro-optical devices wherein the optically thin active regions can have an inhomogeneous permittivity profile.

## Results

### Tunable Metasurface Operating in Transmission Mode

**Figure 2:**
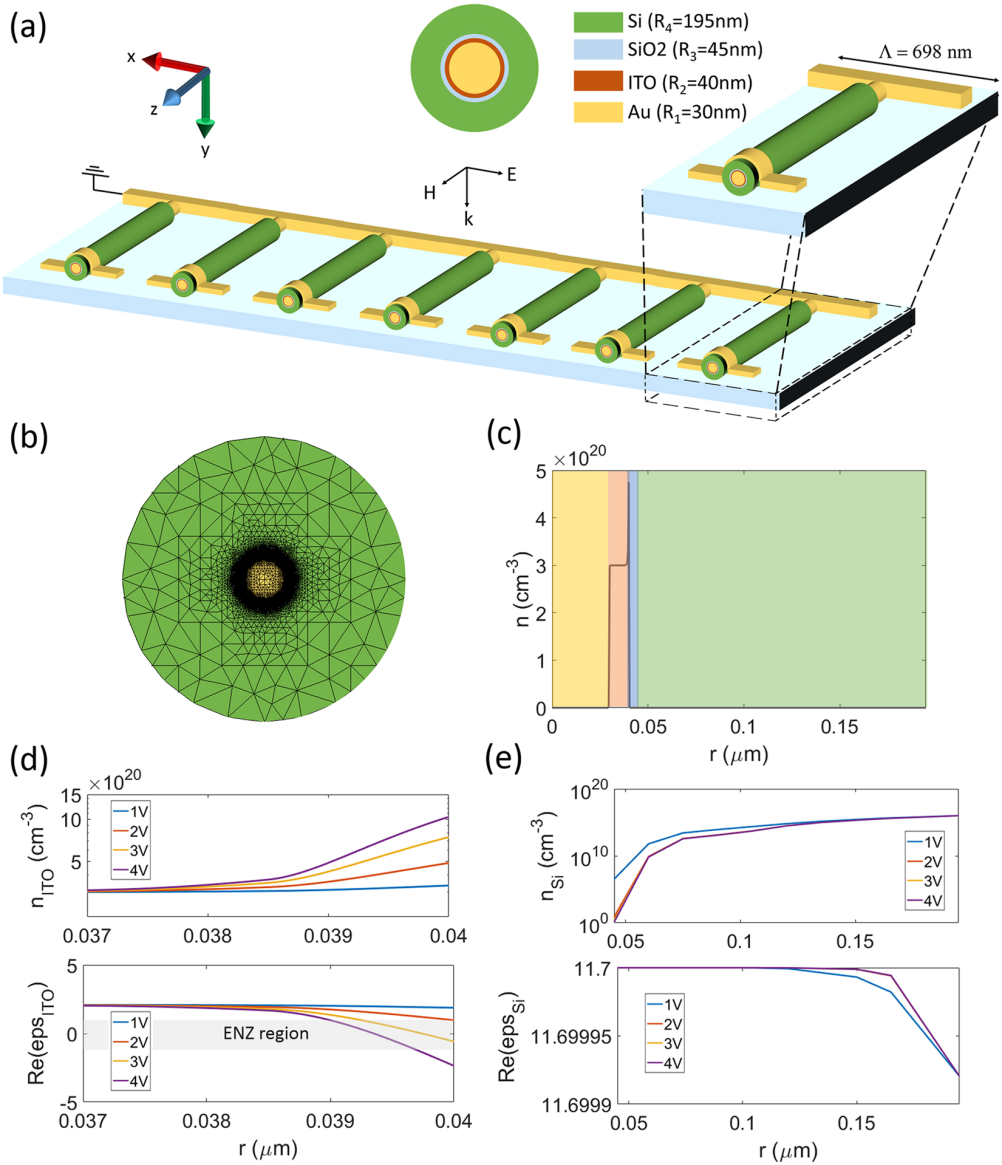
(**a**) The schematic and geometrical parameters of the tunable metasurface operating in the transmission mode. (**b**) The multiscale FEM mesh used for solving drift-diffusion equations. (**c**) Electron concentration distribution in the radial direction for the applied bias of 2 volts. (**d**) Electron concentration and permittivity of ITO corresponding to the operating wavelength of 1550 nm at the interface with the insulator for different applied voltages. (**e**) Redistribution of electron concentration in the silicon layer and its impact on the permittivity of silicon at the operating wavelength of 1550 nm for different applied voltages.

Recently, there have been several works on the demonstration of phase modulation using ITO-based metasurfaces in the reflection mode^23, 27–29^. The proposed structures integrate a thin layer of ITO into metal-insulator-metal (MIM) structures which allows modulation of the reflection phase through critical coupling of ITO active layer into the gap surface plasmon modes. The metallic back mirror in these structures not only makes the biasing of ITO layer feasible, it also provides the enhanced light-matter interaction required for critical coupling. Moreover, the presence of back-mirror helps to obtain larger phase shifts in the reflection. It should be remarked that the reflection efficiency of these structures has been limited due to the restriction of operation at the resonant condition and parasitic losses in the MIM structures. The realization of an ITO-based metasurface for phase modulation in the transmission mode has remained as-yet unrealized. Here, we address this for the first time by integrating ITO into a metal-oxide-semiconductor (MOS) nanowire. We will show that the largest swing in the transmission phase can be achieved by operating at the transmission dip corresponding to the resonance which means similar to the reflection counterparts, the large phase modulation comes at the cost of low amplitude efficiency. The schematic of the proposed metasurface design and its unit-cell is shown in Fig. 2(a). It is composed of an array of subwavelength multimaterial nanowires with a subwavelength period of 698 nm on a SiO_2_ substrate. The cores are made of gold with a radius of 30 nm. Subsequently, very thin ITO and SiO_2_ layers are coated onto the cores with respective radial thicknesses of 10 nm and 5 nm. The outer layer is silicon and the overall radius of the nanowire is 195 nm. The structure is illuminated normally by a plane wave propagating along y axis with transverse-electric (TE) polarization i.e. the magnetic field is along the nanowires axis (TE_*z*_). Due to the high refractive index of silicon, rotation of the electric current can give rise to magnetic resonances while electric resonances cannot be excited as in this case the induced modes inside the nanowires are purely TE (since there is no cross-polarized coupling when the incident wave does not have out-of-plane variation) and the magnetic field cannot form closed loops^44^. The geometrical parameters of the design are chosen such that the structure exhibits a magnetic resonance resonance at the operating wavelength of 1550 nm at no-bias condition. In the core-shell/multimaterial nanoparticles, the filling fraction and shell radius of the particles determine the scattering and resonant properties of the layered system^46, 47^. As such, a parametric study is conducted to determine the required outer radius and filling fraction of the multimaterial nanowire. The results of this study are included in the Supplementary Information. The electrical biasing can be done by applying a voltage between the gold core and the silicon shell which requires doping of the silicon layer. Here, we use a moderately doped n-type silicon with the background concentration of 10^16^ cm^−3^ and the background carrier concentration in the ITO layer is 3 × 10^20^ cm^−3^. Figure 2(b) depicts the multiscale triangular mesh that has been used in Cogenda FEM solver for capturing the multiscale transport features. The gold cores are connected to a ground plane while a positive electrical voltage is applied to the silicon shell. In this way, different phase shifts can be imposed on each element by controlling the bias voltage without the need to change the geometry or orientation of the elements as in the case of previously reported static optical metasurfaces. The gold is modeled with experimentally obtained values for the complex permittivity^48^ while the spatial distribution of complex permittivity in silicon and ITO is obtained via Drude model as a function of carrier concentration and wavelength. The parameters of the Drude model corresponding to each material are brought in the Methods section. The relative permittivities of silicon and ITO are plotted as functions of carrier concentration at the operating wavelength of 1550 mm in the Supplementary Information. It should be noted that silicon is judiciously chosen as the positive contact for biasing. In the case that silicon comes in touch with ITO, the electron redistribution in the silicon layer leads to perturbation of its permittivity profile as well as considerable carrier-induced optical loss (see Supplementary Information for variations of silicon permittivity versus carrier concentration and further discussion).

The distribution of electron concentration is obtained in all regions of the device by solving drift-diffusion equations. A typical demonstration of radial electron distribution over all regions of the device is shown in Fig. 2(c) for the applied bias of 2 V. As it can be seen, the charge concentration is increased at the interface between ITO and the insulator (SiO_2_) forming an accumulation layer with an inhomogeneous profile. The simulation is carried out for different applied voltages. Figure 2(d) demonstrates the electron redistribution and the corresponding permittivity profile in the ITO layer over a radial distance of 3 nm from the interface of ITO-SiO_2_ at the operating wavelength of 1550 nm. Figure 2(e) shows the electron redistribution in the silicon layer with the corresponding permittivity profile at the operating wavelength. The electron concentration in the insulator will be zero due to negligible leakage current (The I–V characteristic of the building block is brought in the Supplementary Information).

The results show that the change in the silicon permittivity due to the electron redistribution is negligible in all cases. This is while the dielectric permittivity of ITO substantially changes over a region within around 2 nm of the ITO-SiO_2_ interface due to the formation of the accumulation layer. The grey area highlights the ENZ region of ITO where the real part of the permittivity is between 1 and −1. Under a positive bias, the real part of the permittivity at the ITO-SiO_2_ interface decreases, reaching the ENZ condition at an applied bias of 2 V. The thickness of the ENZ region reaches its maximum at 0.8 nm for an applied bias of 3 V.

**Figure 3:**
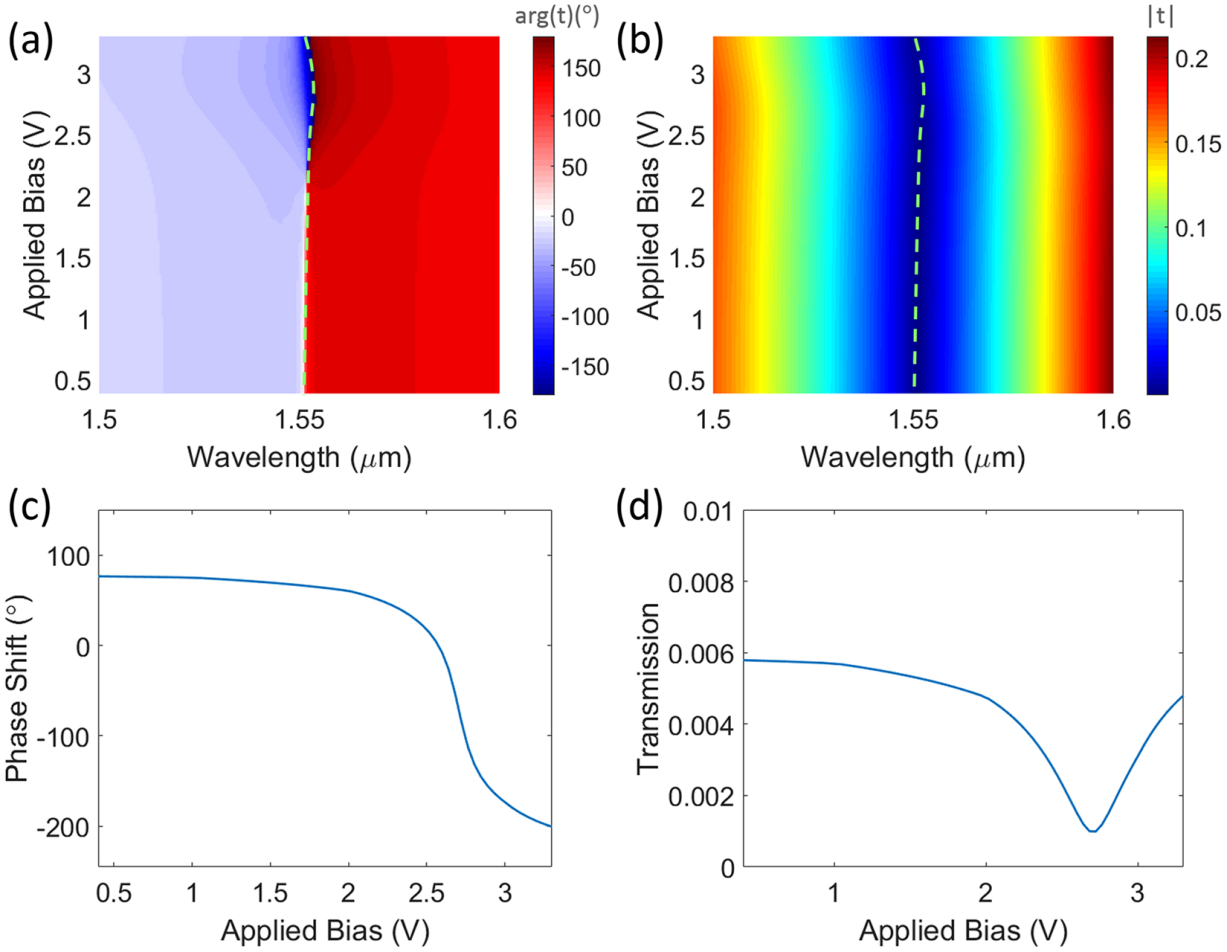
(**a**,**b**) Demonstrate the transmission phase shift and amplitude of the structure as functions of applied bias and wavelength, respectively. The green dashed lines denote the spectral position of the resonance. (**c**,**d**) Depict the transmission phase shift and amplitude as functions of applied bias at the operating wavelength of 1550 nm, respectively.

Figures 3(a,b) demonstrate the transmission phase shift and amplitude spectra as functions of applied bias. An abrupt phase change is achieved in the wavelength spectrum around the transmission dip corresponding to the magnetic resonance. This abrupt phase change enables affording a significant phase modulation at the resonant wavelength through critical coupling to the ENZ region of the ITO layer and shifting the resonance by just a small margin. This is evident from the plots by observing the shift in the resonance and the phase pickup when the applied voltage is increased from 2 V to 3 V, corresponding to the formation of ENZ region. The green dashed lines in Fig. 3(a,b) denote the position of the transmission dip corresponding to the magnetic resonance. As it can be observed, by increasing the applied bias the resonance shifts to longer wavelengths until the relative permittivity of ITO at the ITO-SiO_2_ interface reaches zero (V ≈ 2.7). After this point, increasing the voltage will shift the resonance into shorter wavelengths. This change in the direction of resonant wavelength shift is a result of the change in the optical properties of the accumulation layer from dielectric to metallic. When the applied bias continues to increase, the relative permittivity of accumulation layer crosses zero and becomes negative which makes it to optically behave as a metal, thus shrinking the effective thickness of the dielectric regions (background ITO and silica layers) and shifting the resonant wavelength of the nanowire into shorter wavelengths. Figures 3(c,d) depict the calculated transmission phase shift and amplitude as functions of applied bias at the operating wavelength of 1550 nm, respectively. As it can be observed from the results, a large phase shift of over 280° (≈1.55*π*) is afforded.

**Figure 4:**
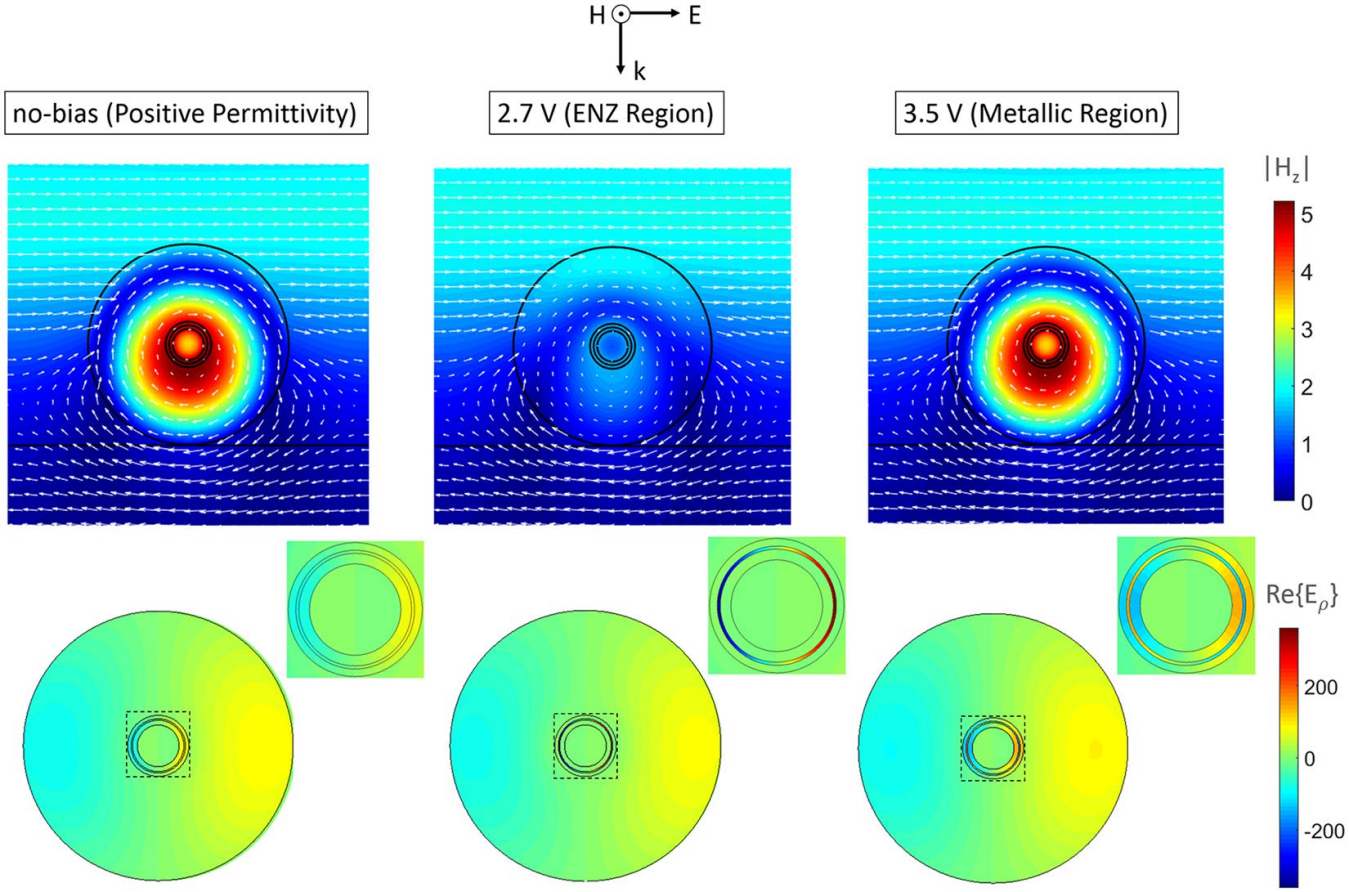
The magnetic field intensity and electric field lines as well as the real part of the electric field normal to the boundaries (E_*ρ*_) inside the nanowire for three cases of no-bias, formation of ENZ region and metallic region. The magnified views show the ITO-SiO_2_ interface.

In order to gain more physical insight to the underlying physical phenomenon of the afforded phase modulation by the ITO-based metasurface and clarify the impact of moving from the positive to negative permittivity, we explore electric and magnetic field profiles inside the nanowire at the operating wavelength of 1550 nm. Figure 4 shows the magnetic field intensity and electric field lines as well as the real part of the electric field normal to the boundaries (E_*ρ*_) inside the nanowire for three cases of no-bias, formation of ENZ region and metallic region. At no-bias, when the relative permittivity of ITO layer is positive, the nanowire is at resonance with the chosen geometrical parameters. The electric displacement current loop and the associated enhanced magnetic field intensity in its center characterizes the magnetic nature of the resonance. The real part of the electric field shows a node at the center corresponding to a magnetic dipole (MD)^49, 50^. By increasing the applied bias to 2.7 V, an ENZ region gets formed inside the ITO layer over a short distance from the ITO-SiO_2_ interface. The magnetic resonance is shifted into longer wavelengths decreasing the magnetic field confinement inside the nanowire at the operating wavelength and deforming the current loop. Moreover, we notice a significant enhancement of E_*ρ*_ inside the ENZ region which can be clearly seen in the magnified view of the ITO-SiO_2_ interface. This enhancement can be justified by observing the continuity of the displacement current component normal to the boundaries which requires stronger electric field confinement to low-permittivity regions. As the applied bias increases, the relative permittivity of ITO accumulation layer continues to decrease toward more negative values and the accumulation layer becomes more and more metallic thus shifting the resonant wavelength into shorter wavelengths. As a result, the nanowire is very close to the resonance at 3.5 V and less change is apparent in the magnetic field confinement and electric field lines comparing to the no-bias case. The nature of the mode is still that of a MD mode, but the real part of E_*ρ*_ shows the metallic behavior of the accumulation layer which results into shrinking the effective thickness of dielectric regions. The observations are consistent with the position of resonant wavelength denoted in Fig. 3(a,b).

As demonstrated, a large phase change (≈280°) can be achieved. This electrically-controllable transmission phase modulation can be used in a myriad of optical applications such as the beam shaping and polarization conversion. In such a design, the proposed configuration is composed of the multimaterial nanowires, each of which can be independently addressed and biased. This can realize on-demand scattering phase profiles for beam steering and beam focusing. In the ideal case, continuous beam steering and focusing require a 360° coverage. However, a satisfactory performance may still be obtained with 280° phase coverage as the phase sampling is discrete and this means introducing small errors in a few samples in a large array. In particular, increasing the size of the metasurface should decrease the error introduced by these samples. Here, we use an array of 200 identical elements to develop an electrically driven dynamic beam steering. The required phase profile for steering the beam with an angle of *θ* is ref. 9:

1 $$\varphi (x)={k}_{0}x\,\sin \,(\theta )$$

**Figure 5:**
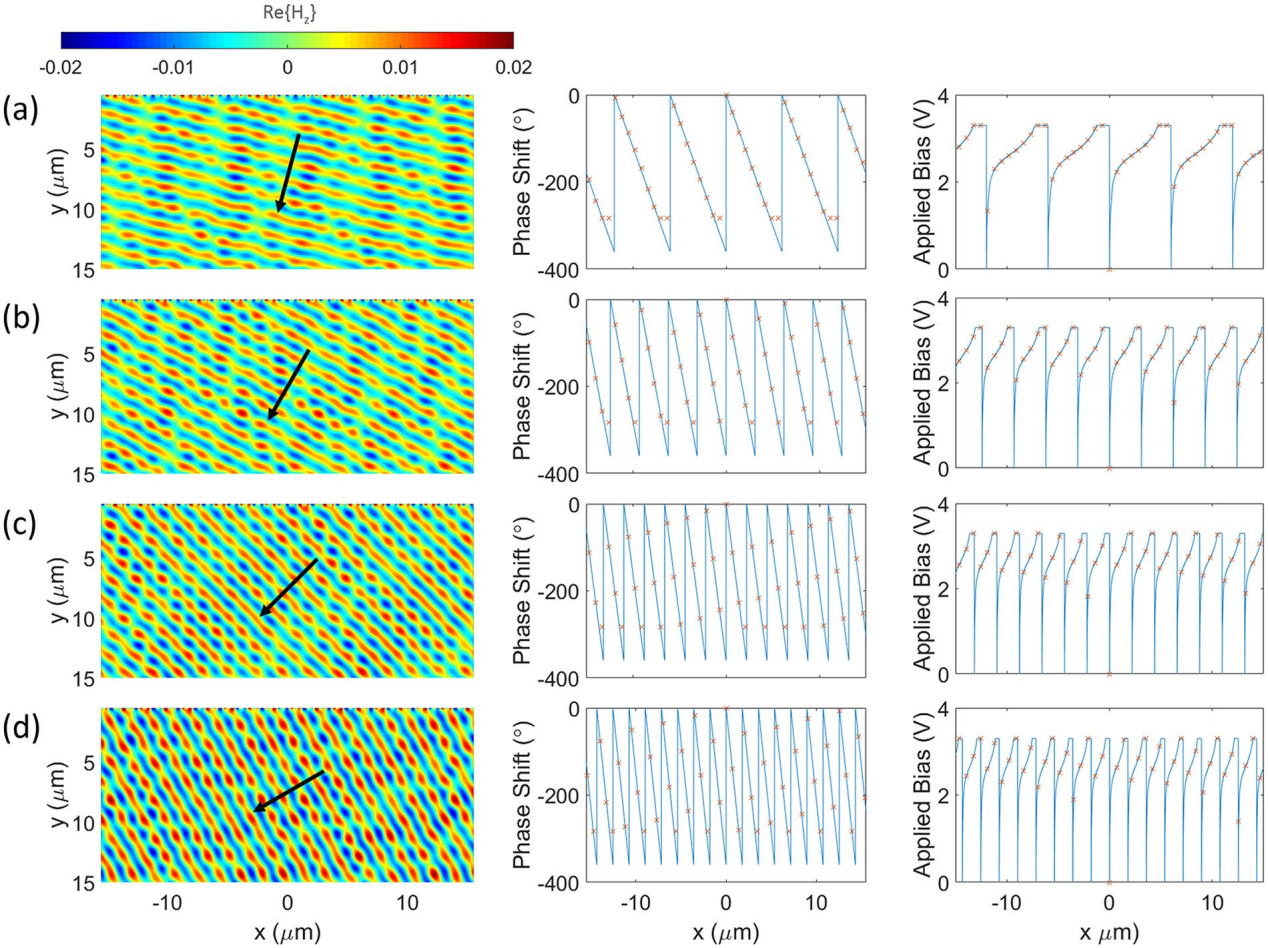
Tunable beam steering. The plane wave is incident normally to the metasurface and is traveling along y axis. (**a**–**d**) Demonstrate the results for the wavefront, required phase shift and applied bias across the metasurface for different steering angles of 15°, 30°, 45° and 60°, respectively. The solid lines denote the continuous required phase shift and applied voltages across the metasurface while “x” markers label the sampling of the profile for each individual element.

This phase profile is realized by applying an external bias independently to each element. Figures 5(a–d) demonstrate the results for the wavefront distribution, required phase shift and applied bias across the metasurface for different steering angles of 15°, 30°, 45° and 60°, respectively. The wavefront plots clearly verify the tunable performance of the metasurface as expected per the design. The above example demonstrates how the active metasurface can tune the functionality and properties of beam steering in real-time. It should be remarked that these structures can also serve multiple roles and realize different functionalities. To illustrate this idea, consider the previous structure, now by applying a different set of voltages we can focus the incident beam and tune the focal length as well. The required phase profile for focusing the beam at a focal length of *f* is given by ref. 7:

2 $$\begin{array}{l}\varphi (x)={k}_{0}(\sqrt{{x}^{2}+{f}^{2}}-f)\end{array}$$

**Figure 6:**
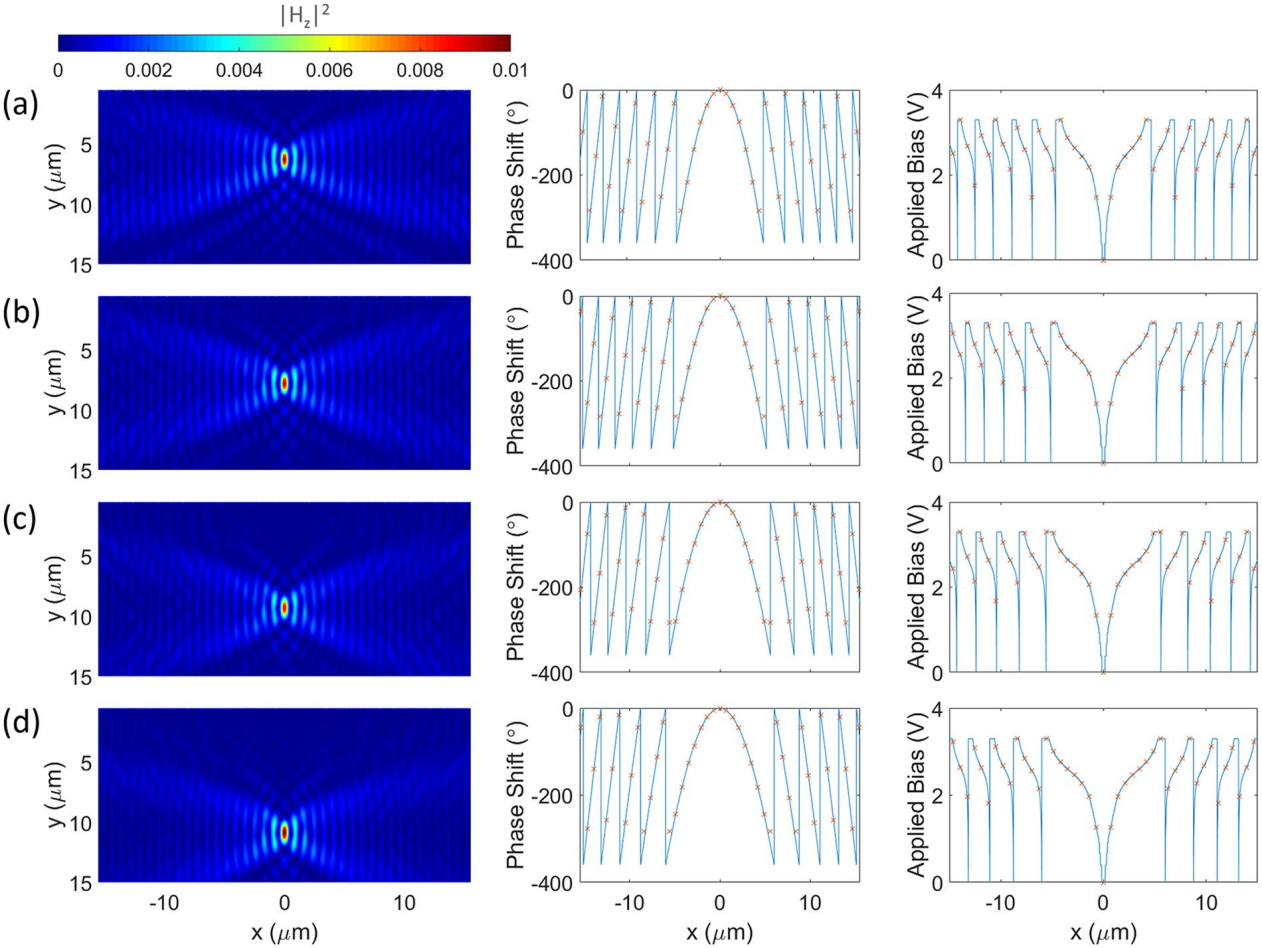
Tunable focusing. The plane wave is incident normally to the metasurface and is traveling along y axis. (**a**–**d**) Demonstrate the results for the field intensity, required phase shift and applied bias across the metasurface for different focal lengths of 4*λ*, 5*λ*, 6*λ* and 7*λ* along y axis, respectively. The solid lines denote the continuous required phase shift and applied voltages across the metasurface while “x” markers label the sampling of the profile for each individual element.

Figures 6(a–d) demonstrate the results for the field intensity, required phase shift and applied bias across the metasurface for different focal lengths of 4*λ*, 5*λ*, 6*λ* and 7*λ* along y axis, respectively. These nearfield plots clearly verify the tunable performance of the metasurface and the location of focal spots are in excellent agreement with the values set by the design.

The amplitude efficiency of the proposed metasurface is limited by the necessity to operate at the resonance for obtaining the largest swing in the phase shift. This has been also the case for previously reported metasurfaces for reflection phase modulation based on ITO^23, 29^ and graphene^24^. It should be remarked that despite the large reflection efficiency of the proposed structure, the reflection phase cannot be modulated as the abrupt change in the spectral phase shift only occurs at the dip of the transmission and the reflection phase shift has a smooth change at the peak. This is demonstrated and discussed in further details in the Supporting Information. It is also noteworthy that as the new tunable materials with lower optical loss become available in the future, there may be the possibility of operating off-the resonance or simultaneous excitation of multiple resonances to improve the amplitude efficiency and obtain the full phase shift (similar to the Kerker-type scattering condition in all-dielectric metasurfaces^49, 50^). However, these scenarios are hindered by the relatively high optical losses in the ITO material. We have identified a route for improving the amplitude efficiency by almost 10 times through using asymmetric bi-nanowires as the constituent elements of the metasurface. The geometrical parameters and the comparison of the performance for this alternative design are brought into the Supplementary Information. Moreover, the possibility of designing a metasurface with full phase shift and very high transmission is investigated in Supplementary Information using a hypothetical low-loss material with the field-effect modulation. It should be noted that due to causality, any physical realization of an ENZ material will be lossy and dispersive, however, with the continuous trend in the discovery of new, high performance plasmonic materials, it is expected that alternative materials with lower loss become available in the future^51, 52^.

Another important limitation in the ITO-based metasurfaces is the narrow bandwidth at which the large phase modulation can be achieved. This narrow bandwidth implies that the operating wavelength at which the maximum phase modulation can be achieved has a critical dependence on the geometrical parameters such that the slightest changes in the geometrical parameters can shift the operating wavelength of the periodic structure. This can devastate the performance of a graded metasurface design with a certain functionality due to the structural non-uniformities which are inevitable in the fabrication process. The dependence of the operating wavelength of the periodic arrangement to the geometrical parameters of the nanowires is studied in the Supplementary Information. Moreover, the cumulative effect of structural non-uniformity on the functionality and performance of the metasurface is investigated comprehensively by considering different levels of random deviations from the original design parameters. It is observed that the functionality is almost preserved while the performance efficiency deteriorates significantly as the geometrical deviations with respect to the original design become larger. Thanks to the recent advances in the drawing and lithography techniques, the minimum feature size of core-shell/multimaterial nanowires has been pushed into sub-5 nm scale^35^, allowing for a satisfactory performance of the ITO-based designs with current state of fabrication technologies.

### Tunable Gradient Index Metamaterials

Another exciting and as-yet unaddressed opportunity that can be offered by ITO is adding tunability and multifunctionality to graded index metamaterials. The concept of digital metamaterials has been recently introduced to synthesize an electromagnetic metamaterial with a desired permittivity, using building blocks composed of only two elemental materials, called ‘metamaterial bits’, possessing two different permittivity functions^5^. This methodology is an ideal route for realization of graded index metamaterials as one may not always have access to all materials with all the required parameter values^45^.

This has been made possible by virtue of internal homogenization^53^, which states that a deeply subwavelength element with different internal inclusions can be homogenized with an effective refractive index which will exhibit a similar scattering performance. Here, inspired by the same idea, we investigate how formation of an accumulation layer in an ITO layer integrated into a deeply subwavelength multimaterial nanowire (with a radius much smaller than the operating wavelength) through an external bias can yield strong variations in the effective refractive index of the nanowire. The approach devised here can be used to add electrical tunability and multifunctionality to metamaterials and photonic crystals.

**Figure 7:**
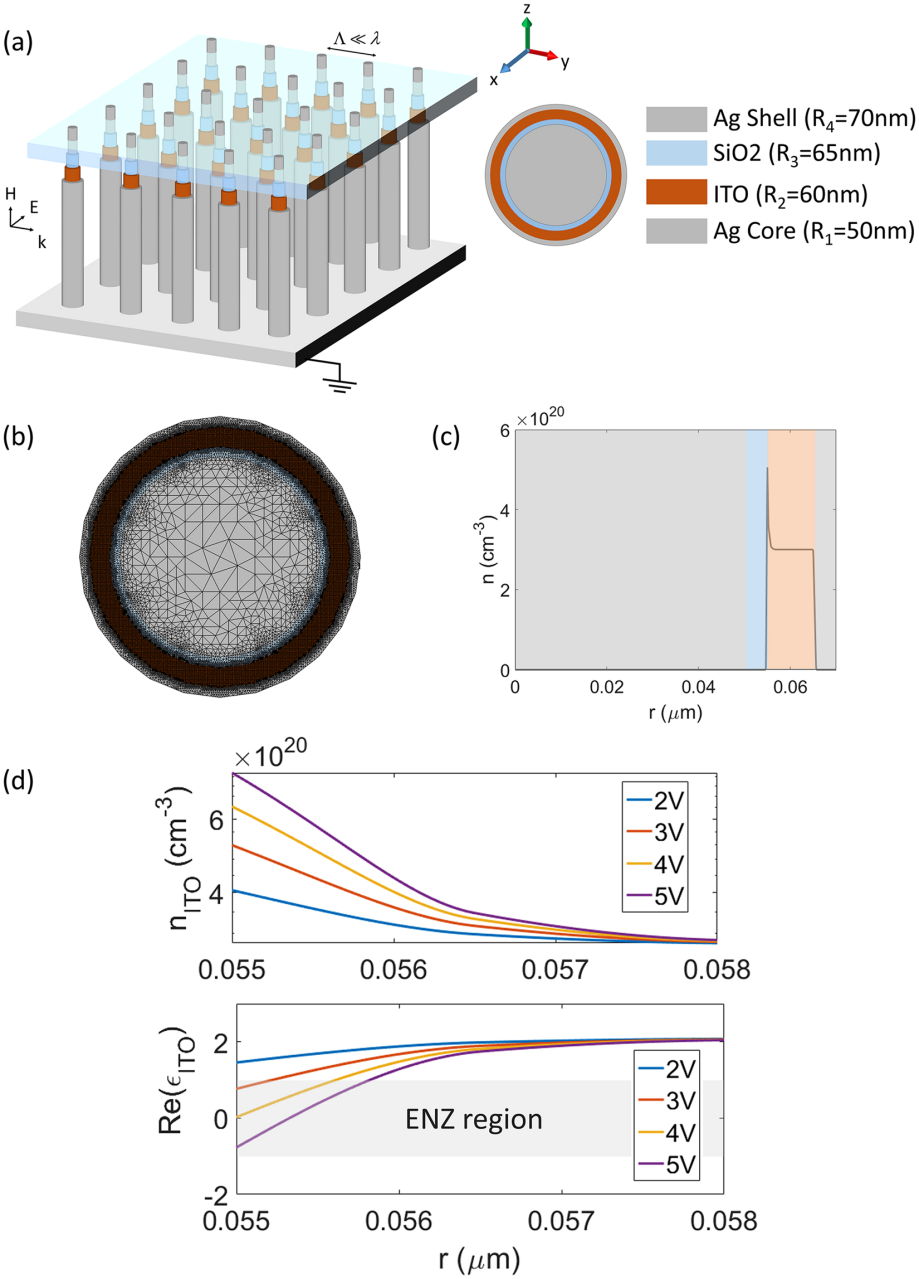
(**a**) The schematic and geometrical parameters of the tunable graded-index metamaterial. (**b**) The multiscale FEM mesh used for solving drift-diffusion equations. (**c**) Electron concentration distribution in the radial direction for the applied bias of 3 volts. (**d**) Electron concentration and permittivity of ITO corresponding to the operating wavelength of 1550 nm at the interface with the insulator for different applied voltages.

The topology of the multimaterial nanowire is chosen in accordance to the biasing requirement. An ITO layer is added into a metal-insulator-metal (MIM) nanowire structure. The geometrical parameters are judiciously chosen to maximize the variations of the effective refractive index of the nanowire at the operating wavelength of 1550 nm. Figure 7(a) demonstrates the schematic of such tunable metamaterial and its unit-cell. The core is made of silver with a radius of 50 nm. Subsequently, very thin SiO_2_ and ITO layers are coated onto the cores with respective radial thicknesses of 5 nm and 10 nm. A silver cladding with the thickness of 5 nm is considered as the outer layer which is not only essential to the design but is also advantageous in terms of fabrication and field-driven alignment of nanowires. The biasing can be done by connecting the metallic shell into an underlying ground plane while the metallic cores can be addressed and biased independently through a biasing grid from the top^37, 38^. The structure is illuminated from the side by a normal plane wave with TE polarization (the magnetic field is along the nanowires axis). The background carrier concentration of the ITO layer is 3 × 10^20^ cm^−3^. Figure 7(b) depicts the multiscale triangular mesh that has been used in Cogenda FEM solver for capturing the multiscale transport features. The shell is connected to the ground plane and a positive voltage is applied to the core. The silver is modeled with experimentally obtained values for complex permittivity^48^ and the spatial distribution of the complex permittivity inside the ITO layer is obtained via Drude model as a function of wavelength and carrier concentration.

The distribution of electron concentration is obtained in all regions of the device by solving drift-diffusion equations. A typical demonstration of radial electron distribution over all regions of the device is shown in Fig. 7(c) for the applied bias of 3 V. As it can be seen, the charge concentration is increased at the interface between ITO and the insulator (SiO_2_) forming an accumulation layer with inhomogeneous profile. The simulation is carried out for different applied voltages (The I–V characteristic of the building block is provided in the Supplemental Information). Figure 7(d) demonstrates the electron redistribution and the corresponding permittivity profile in the ITO layer over a radial distance of 3 nm from the interface of ITO-SiO_2_ at the operating wavelength of 1550 nm. Similar to the case of MOS nanowire considered above, the dielectric permittivity of ITO substantially changes over the region within around 2 nm of the ITO-SiO_2_ interface due to the formation of the accumulation layer. The grey area denotes the ENZ region of ITO where the real part of the permittivity has values between 1 and −1. Under a positive bias, the real part of the permittvity at the ITO-SiO_2_ interface decreases, reaching the ENZ condition at an applied bias of 3 V. The thickness of the ENZ region reaches its maximum at 0.8 nm at 5 V.

**Figure 8:**
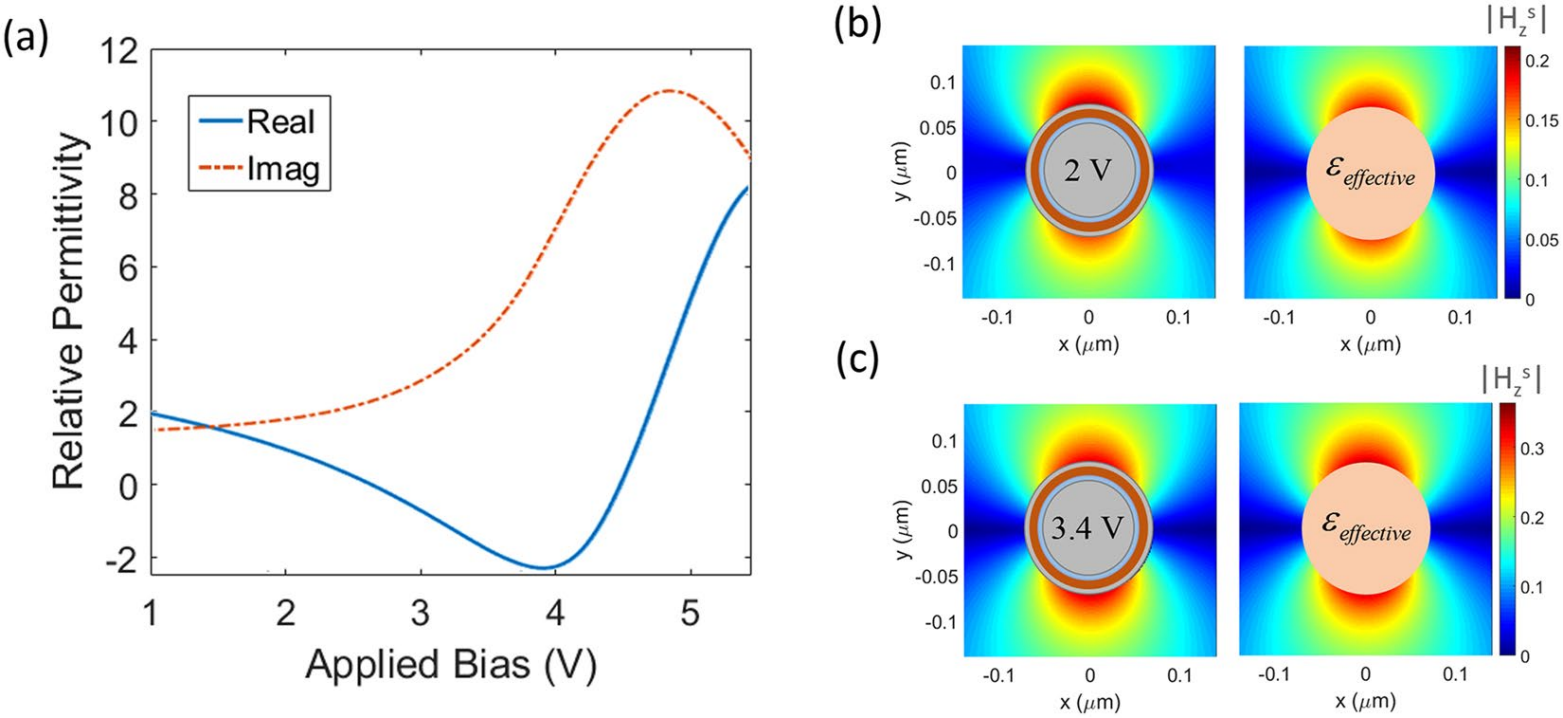
(**a**) The variation of the effective relative permittivity vs applied voltage. (**b**,**c**) Compare the nearfield distribution for the multimaterial nanowires and their homogenized counterparts for applied voltages of 2 and 3.4 volts, respectively. A great agreement can be seen between the results.

In order to determine the effective permittivity of the multimaterial nanowires under study and internally homogenize them, we can either apply the analytical formula for the effective permittivity of a core-shell recursively from inner to outer layers^53^ or use a more general method based on parameter retrieval^54^. The former approach may lead to inaccurate results when the dimensions poorly satisfy the small-radii approximation while the latter is more general and valid regardless as long as the element is in the effective regime. As such, here we have used a parameter retrieval approach to homogenize the multimaterial nanowires under different applied biases. Figure 8(a) demonstrates the obtained results for the real and imaginary parts corresponding to the effective permittivity of the multimaterial nanowires as a function of applied bias to the core. The behavior of effective permittivity versus the applied bias shows a Lorentzian profile which covers a large range of relative permittivity from −2 to 8.

It should be remarked that despite the large imaginary parts obtained for the effective permittivity, the design approach devised in this section is more tolerable to the loss effects comparing to the ITO-based metasurfaces. One reason is that unlike the ITO-based metasurfaces, the physical phenomenon here does not rely on the enhanced light-matter interactions making the role of loss, less crucial. Moreover, in the design procedures based on effective medium theory (EMT) to realize a gradient refractive index, the material loss can always be compensated by using a minimal filling fraction for the lossy material.

For the verification of the internal homogenization procedure, we compare the nearfield distribution of the scattered magnetic field corresponding to the multimaterial nanowires and their homogenized counterparts for different applied biases of 2 V and 3.4 V. The results are shown in Fig. 8(b,c), respectively. An excellent agreement is observed between the scattered field profiles implying that the homogenized nanowires indeed behave similar to the multimaterial counterparts as viewed from outside. It should be noted that the field profiles inside the nanowires may differ for the multimaterial and homogenized counterparts.

**Figure 9:**
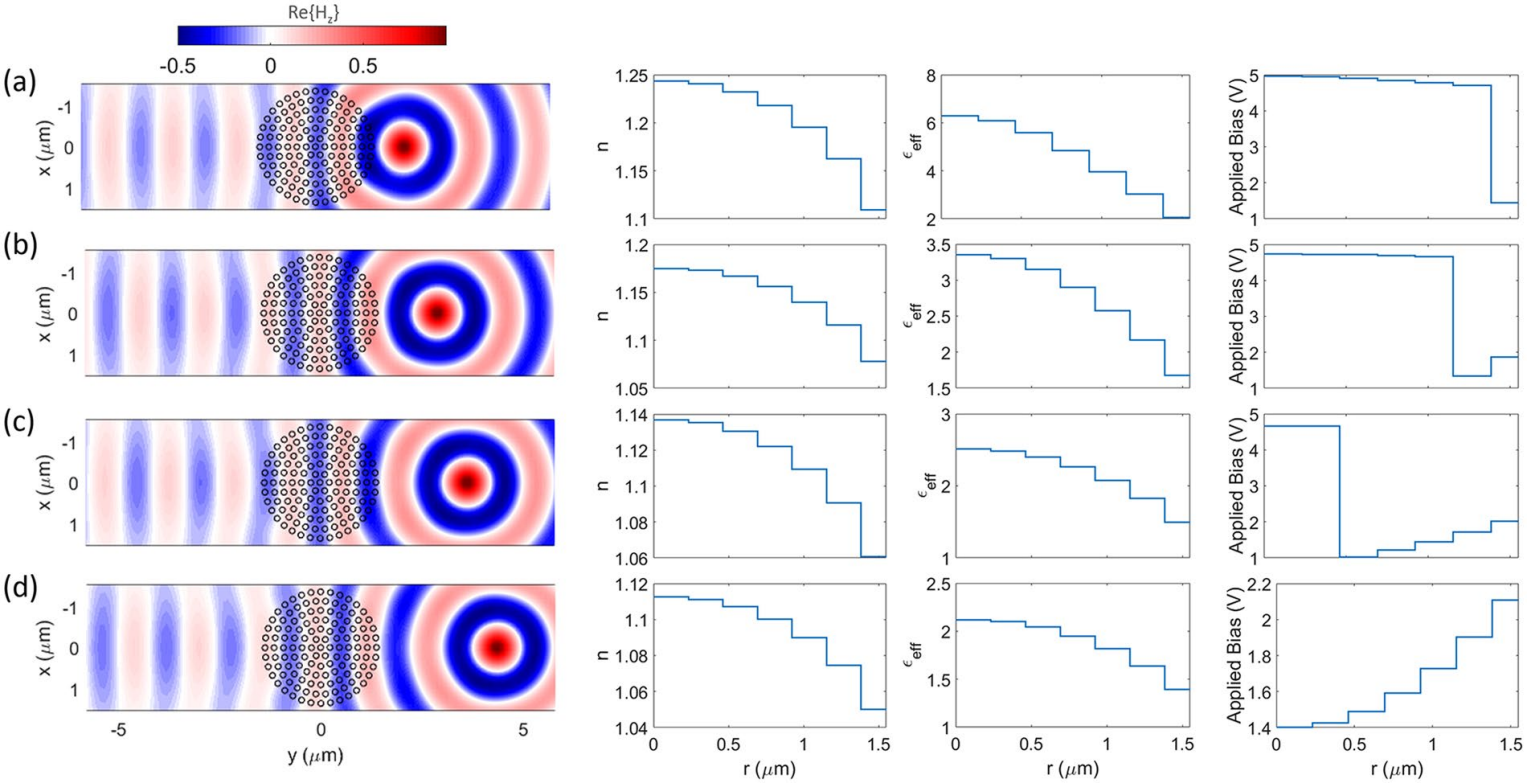
Tunable Luneburg Lens for a defocused source located at the right side. The spacing between the layers is 160 nm (≈*λ*/10) which is within the effective medium region. (**a**–**d**) Demonstrate the results of the wavefronts, required refractive index, effective permittivity and applied bias across the radial direction of the metamaterial for different focal lengths of 1.5*R*_*lens*_, 2*R*_*lens*_, 2.5*R*_*lens*_ and 3*R*_*lens*_, respectively. The wavefronts leaving the lens are planar demonstrating tunable beam collimating. The structure can also be used to concentrate the incoming plane waves with a tunable focal length.

We apply this methodology to develop a generalized Luneburg lens with electrically tunable focal length. A Luneburg lens is a cylindrically symmetric lens which is capable of mapping the rays coming from a point source on the surface of the lens into a collimated beam on the diametrically opposite side of the lens. However, in most of the practical applications, a point source cannot be put on the surface of the lens. In order to address this challenge, a generalized Luneburg lens can be used which is capable of collimating the beams coming from a point source located at some distance away outside the lens. The required refractive index profile for the generalized Luneburg lens is given by ref. 55:

3 $$n(r)=\exp (w(r,a))$$

in which:

4 $$w(r,a)=\frac{{R}_{lens}}{\pi }{\int }_{r}^{{R}_{lens}}\,\frac{{\sin }^{-1}\,(t/a)}{\sqrt{{t}^{2}-{r}^{2}}}dt$$

where *R*_*lens*_ is the radius of the lens and *a* is the distance of the point source from the center of the lens. According to the EMT, in order to realize this refractive index profile using a piecewise-constant approximation and a set of multimaterial nanowires, the required effective permittivity for each nanowire can be obtained as ref. 8:

5 $${\varepsilon }_{eff}={\varepsilon }_{host}\frac{ff({n}^{2}+{\varepsilon }_{host})+({n}^{2}-{\varepsilon }_{host})}{ff({n}^{2}+{\varepsilon }_{host})-({n}^{2}-{\varepsilon }_{host})}$$

where *ε*_*host*_ is the host medium permittivity, *ff* is the filling fraction and *n* is the required refractive index to be realized. The filling fraction can be obtained by dividing the total area of the lens by the total area occupied by nanowires. Here, we consider a circular array configuration consisted of 127 identical nanowires arranged in 7 layers with an edge-to-edge separation distance of 90 nm forming a circular lens with a diameter of *D*_*lens*_ = 2*λ* = 3100 nm. The filling fraction in this case is *ff* = 0.296 and the sampling period in the radial direction is 160 nm (≈*λ*/10) which is within the effective medium region. Using equations 3–5 and according to Fig. 8(a), we can address and bias each nanowire independently to realize a certain spatial distribution for the refractive index to tune the focal length of the Luneburg lens. The wavefronts and the required radial distribution of refractive index, effective permittivity and applied bias are shown in Fig. 9(a–d) for the focal lengths of 1.5*R*_*lens*_, 2*R*_*lens*_, 2.5*R*_*lens*_ and 3*R*_*lens*_, respectively. In the cases where more than a choice for biasing voltage were applicable, the one resulting to lower loss was chosen. The plots clearly verify the tunable performance of the lens, showing planar wavefronts leaving the lens for sources placed at varying distances from the lens. As it can be observed from the results, the effect of material loss do not appreciably degrade the lens performance as the loss is compensated by the spacing between the nanowires. Moreover, the field attenuation through the lens is limited as the light does not interact strongly with the material. The designed lens can also be used to concentrate incoming plane waves at the opposite side with a tunable focal length. In order to quantize the performance of the finite-sized aperiodic lens, we define the collimation efficiency as the ratio of optical power intensity of the collimated wavefront to the power intensity of the cylindrical wavefront in the absence of the lens. The obtained efficiencies for different cases presented in Fig. 9(a–d) are 78.94%, 82.84%, 88.88% and 96.09%, respectively.

In summary, we presented novel designs for electrically tunable graded metasurfaces and metamaterials by incorporating ITO into multimaterial nanowires with MOS and MIM configurations. We demonstrated phase modulation of the transmitted light using a flat ITO-based metasurface which can be used for various applications such as dynamic holograms, tunable beam steering and focusing lens. Moreover, we showed the field modulation effect in ITO can be used to tune the effective refractive index of deeply subwavelength multimaterial nanowires enabling tunability of graded index metamaterials with many potential applications for tunable focusing, steering, guiding and routing of light. The electro-optical phenomenon was characterized rigorously by linking transport and electromagnetic models through a hierarchical multiscale approach by translating the spatial distribution of carrier concentration in active regions to the complex-valued permittivity via Drude model and subsequently supplying it to the electromagnetic model.

It should be remarked that the proposed structures are within reach of fabrication with the current state of drawing and lithography techniques^35, 36^ and the fabrication feasibility of similar multimaterial configurations has been shown experimentally for various applications such as energy harvesting^56, 57^, biosensing^58^ and electronic devices^37–40^. These techniques have allowed for processing next generation field-effect transistors incorporating new material compositions with multiple source/drain contacts in highly dense and well-ordered vertically or laterally oriented nanowire arrays on substrates.

## Methods

The rigorous characterization of electro-optical phenomena requires a multiscale and multiphysics approach. A simulation of active electro-optical devices does not only need to solve drift-diffusion equations to characterize the charge transport in the active regions of the device but also to obtain the electromagnetic response of the structure by solving Maxwell’s equations. Moreover, the dimensions of active regions of the device are in the nanometer-scale while possessing gradient features whereas the overall dimensions of the device can be in order of micron. As such, a reliable simulation of the device requires linking between different models and resolving different length scales in the device with high accuracy.

In this work, we have used the open-source Cogenda device simulator^41^ which is a finite-element based drift-diffusion solver. The triangular meshing is used to discretize the problem space and the mesh is refined in the active regions to capture the multiscale features with a high accuracy and efficiency. The ITO material is modeled as a semiconductor with the DC permittivity of 9.3^32^, bandgap of E_*bg*_ = 2.8 eV^59^, electron affinity of *χ* = 5 eV, electron mobility of 38 cm^2^/(V.s) and effective electron mass of m* = 0.25 m_*e*_^23, 60^. In order to do this, it is necessary to modify the material library source code.

The electron distribution is then obtained in the active regions of the electro-optical device. Subsequently, the spatial distribution of the electron is translated to the spatial distribution of the complex permittivity via Drude model. For this purpose, we have refs 23 and 60:

6 $$\varepsilon (\omega )={\varepsilon }_{inf}-\frac{{\omega }_{p}^{2}}{{\omega }^{2}+i\omega {\rm{\Gamma }}}$$

where *ε*_*inf*_ is the high frequency permittivity, and *ω*_*p*_ and Γ are plasma frequency and damping constant, respectively given by:

7 $${\omega }_{p}^{2}=\frac{n{e}^{2}}{{\varepsilon }_{0}{m}^{\ast }}$$

8 $${\rm{\Gamma }}=1/\tau =\frac{e}{\mu {m}^{\ast }}$$

where *n* is the carrier concentration, *e* is the electron charge, *ε*_0_ is the vacuum permittivity, m* is the effective electron mass, *τ* is the scattering time and *μ* is the electron mobility. For ITO we have *ε*_*inf*_ = 3.9, m* = 0.25*m*_*e*_ and *μ* = 38 cm^2^/V.s^23^ which lead to Γ = 185 THz and *ω*_*p*_ = 1.65 rad.PHz for a carrier concentration of *n* = 3 × 10^20^ cm^−3^. It should be noted that upon the application of electrical bias the carrier concentration in the charge accumulation layer within ITO may be increased up to *n* = 9 × 10^20^ cm^−3^ causing the plasmon frequency to increase up to *ω*_*p*_ = 2.86 rad.PHz. For silicon *ε*_*inf*_ = 11.7, m* = 0.27*m*_*e*_ and *μ* = 80 cm^2^/V.s^52^ which result into Γ = 180 THz and *ω*_*p*_ = 10.85 rad. THz for a background carrier concentration of *n* = 10 × 10^16^ cm^−3^.

Once the complex permittivity is obtained in all active regions of the device, it can be supplied into an electromagnetic model for characterization of the optical response. In this work, we utilize a robust aggregate transition matrix (T-matrix) approach^42^ for electromagnetic modeling of substrate-supported arrays of multilayer inhomogeneous nanowires^43–45^. Owing to its superior efficiency compared to brute-force computational methods, the T-matrix model facilitates the performance characterization of large-scale electro-optical devices wherein the optically thin active regions can have an inhomogeneous permittivity profile. For modeling the radially inhomogeneous permittivity profiles, we adopt a finely stratified approach^61^ which has been shown to have an excellent agreement with the exact inhomogeneous profile^62^. Such a modeling approach which involves passing a parameter from one discipline to another is referred to as a hierarchical multiscale modeling^63^.

## Electronic supplementary material

Supplementary Information
