# Supplementary material for: Electrically Tunable Metamaterials Based on Multimaterial Nanowires Incorporating Transparent Conductive Oxides
Source: Sci Rep. 2017 Aug 30;7:10055. doi: 10.1038/s41598-017-09523-4 (PMC5577228; doi:10.1038/s41598-017-09523-4)
Supplement: Supplementary file 1 — Supplementary Information [file 41598_2017_9523_MOESM1_ESM.pdf]

Supplementary Information: Electrically Tunable Metamaterials Based on Multimaterial Nanowires Incorporating Transparent Conductive Oxides

Mohammad Mahdi Salary[1] **and Hossein Mosallaei**[1,*]

[1]Metamaterials Laboratory, Northeastern University, Electrical and Computer Engineering Department, Boston, MA 02115, USA
[*]hosseinm@ece.neu.edu

ITO and Silicon Permittivities vs Carrier Concentration

The relative permittivities of ITO and an n-type silicon as a function of carrier concentration are calculated via Drude model using the parameters given in the manuscript and are plotted in Fig. S1(a) and (b), respectively at the operating wavelength of 1550 nm. As it can be seen, the real part of ITO permittivity changes its sign from positive to negative and the material loss increases as the carrier concentration is increased form 3×10^{20} cm^{-3} to 8×10^{20} cm^{-3}. For the silicon, the material loss and the change in the real part are negligible up to a carrier concentration of 10^{19} cm^{-3}. After this point, increasing the carrier concentration induces significant changes in the real part of the permittivity. Moreover, the carrier-damping loss is considerably increased.

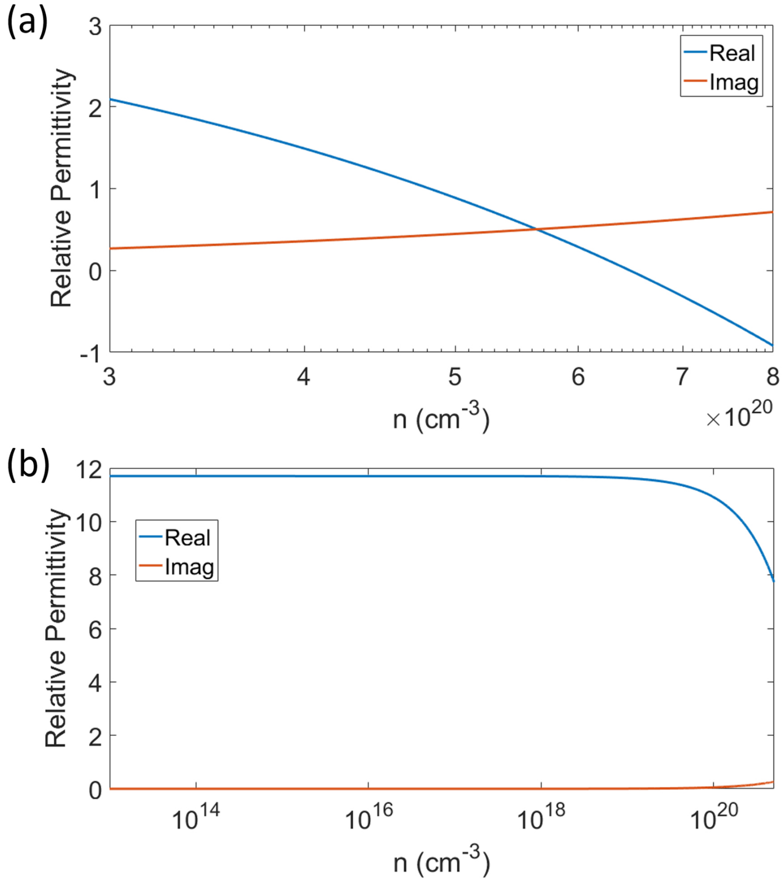

Figure S 1. Relative permittivities of (a) ITO and (b) silicon as a function of carrier concentration.

The behavior of silicon permittivity as a function of carrier concentration suggests that in order to avoid material losses and

a perturbation in the permittivity profile of silicon, the silicon should not be used as a negative contact for biasing. It should be noted that in this case the radial order of ITO and insulator layers should be interchanged to achieve charge accumulation at the ITO-SiO_2 interface. This causes the doping impurity to spread from ITO to the silicon, significantly increasing the carrier concentration in the silicon as they come in contact. Figure S2 demonstrates a typical case where an n-type silicon with the

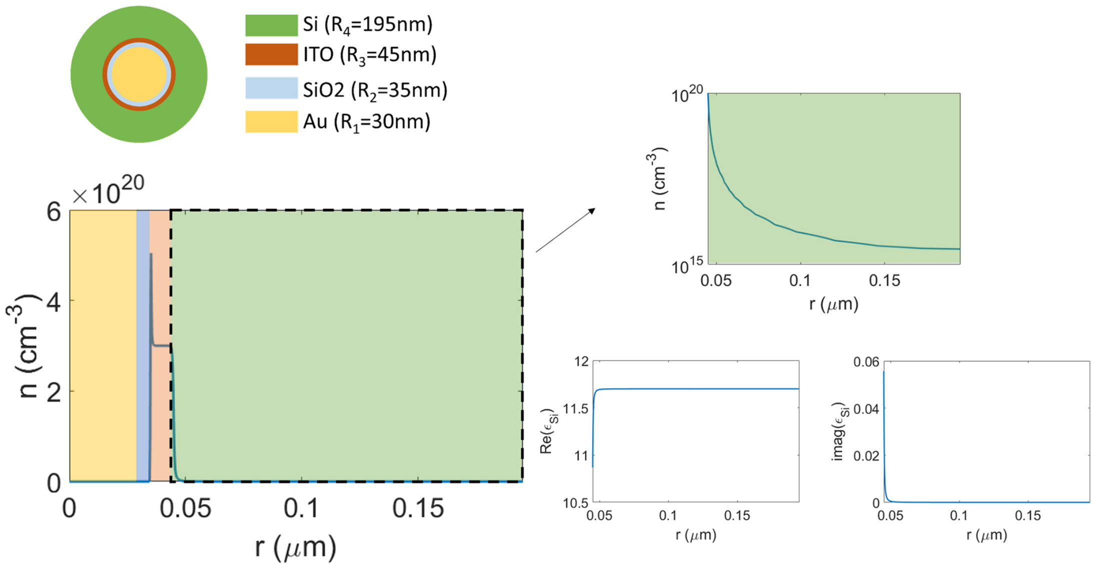

Figure S 2. The electron redistribution inside the MOS nanowire when an n-type silicon with a background concentration of 10^{16} cm^{-3} is used as negative contact for electrical biasing under the applied bias of 3 V. The magnified view demonstrates the redistribution of the carrier concentration inside the silicon layer. The real and imaginary part of the silicon permittivity corresponding to the carrier concentration is plotted in below which demonstrate the perturbation in the real part and carrier-induced optical loss.

background carrier concentration of 10^{16} cm^{-3} is used as the negative contact for biasing and a positive voltage of 3 V is applied to the gold core. The background carrier concentration of the ITO layer is 10^{20} cm^{-3}. The redistribution of electron concentration in the silicon layer is magnified and it can be observed that the carrier concentration at the silicon-ITO interface is 10^{20} cm^{-3} which decreases exponentially across the layer down to 10^{16} cm^{-3} causing a perturbation in the permittivity profile and inducing optical losses in the silicon layer.

Critical Coupling Condition in the Metasurface

As it was mentioned in the manuscript, the largest swing in the transmission phase shift can be obtained by operating at the transmission-dip corresponding to the resonance which limits the transmission amplitude efficiency. In order to gain more insight to this limitation, the amplitude and phase spectra of the transmission and reflection of the transmitting metasurface presented in the manuscript are plotted in Fig. S3. The results show that increasing the applied bias causes relatively small shifts in the resonance wavelength. As such, to achieve a large phase pickup, an abrupt phase change is required in the resonance. This condition is only satisfied at the transmission-dip while the reflection phase exhibits a smooth change at the peak corresponding to the resonance. This is the reason behind the large afforded phase modulation in the transmission while the reflection does not pick up any significant phase shift which hinders performance of the proposed metasurface in the reflection mode.

Assessment of Core-Shell Ratio for Achieving Critical Coupling Condition

The core-shell ratio has a critical role in achieving critical coupling condition at the desired operating wavelength. This ratio is chosen by performing a careful parametric study. Figure S4 shows the results for the transmission amplitude and phase at the operating wavelength of λ=1550 nm as a function of shell radius and filling fraction of the metallic core in the multimaterial nanowire element. Two TE resonant modes can be identified corresponding to magnetic dipole mode (1st mode) and a higher order magnetic mode (2nd mode) which are denoted by blue and red dashed lines. The resonant modes merge together by increasing the filling fraction (as denoted by the white dashed line in the figure). Despite the high transmission efficiency at the merged resonances, the phase shift become smooth and no significant phase modulation can be obtained by the slight shift of the

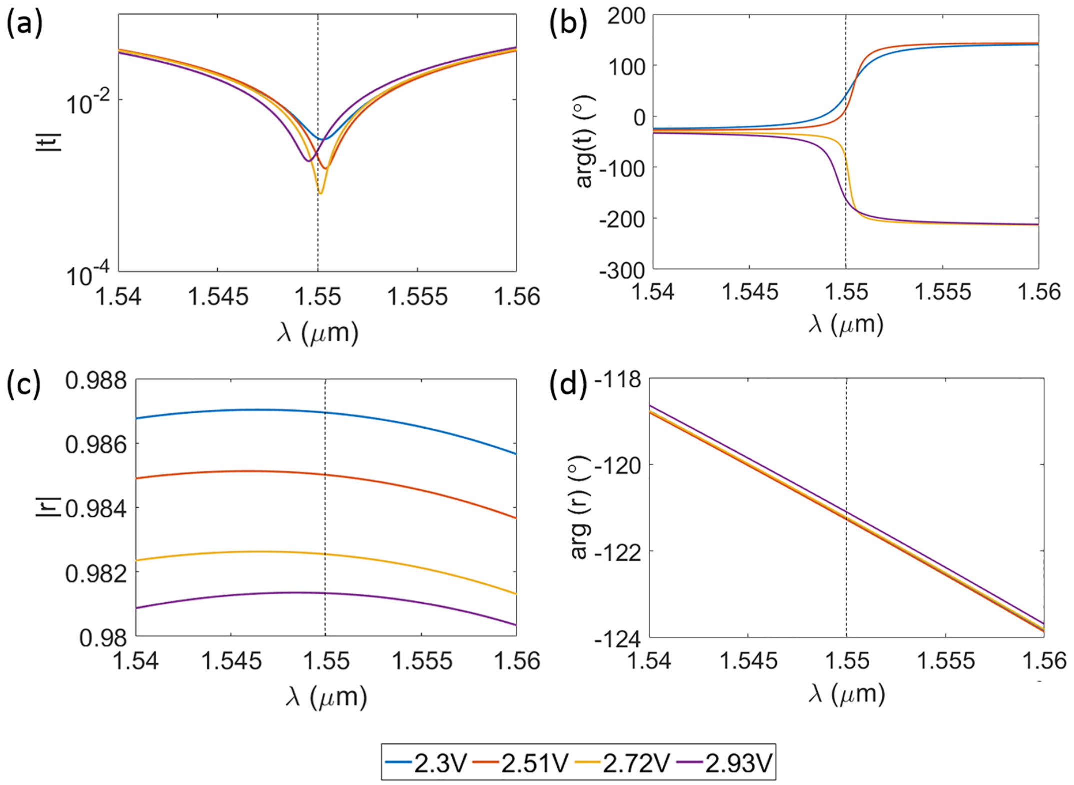

Figure S 3. (a) and (b) demonstrate the transmission amplitude and phase shift spectra for different applied bias corresponding to the metasurface design presented in the manuscript, respectively. (c) and (d) demonstrate the same corresponding to the reflection.

resonance using the field-modulation effect of ITO. Moreover, operating at the second resonance does not give significant phase modulation as well, since the spectral phase shift in this case corresponding to the unbiased case has a decaying profile and cannot be flipped by coupling to the ENZ region of ITO accumulation layer. As such, the only working design can be achieved by operating at the first resonant mode. According to Fig. S4, several geometrical parameters can be chosen which lead to same amplitude and phase modulation efficiency. The parameters corresponding to the design presented here (R_{Shell}=195 nm, Filling Fraction=0.154) are denoted in the figure by the ”x” marker.

Evaluation of Metasurface Performance with respect to Structural Non-uniformity

As it can be seen from Fig. S3b, the afforded phase modulation is limited to a very narrow bandwidth and as the wavelength is increased or decreased the phase modulation decreases. This is one of the limitations in ITO-based metasurfaces which is attributed to the critical coupling condition.

This also implies that changing the geometrical parameters slightly can shift the resonant wavelength to higher or lower wavelengths and limiting the performance at a fixed operating wavelength as a result. Figure S5 shows the dependence of resonant wavelengths on the geometrical parameters of the unit-cell by plotting the spectral transmission amplitude and phase as functions of shell radius for a fixed filling fraction (=0.154) and as a function of filling fraction for a fixed shell radius (=195 nm). The parameters corresponding to the design here (λ=1550nm, R_{Shell}=195 nm, Filling Fraction=0.154) are shown by ”x” marker in the figure. In order to evaluate the effect of structural non-uniformity on the performance of the metasurface, we consider the focusing functionality at a focal distance of 7λ shown in the manuscript and quantize the performance in terms of focusing efficiency which is defined as the ratio of the optical power in the focal spot to the transmitted optical power. We randomly perturb the filling fractions and shell radii of the metasurface elements (consisted of 200 multimaterial nanowires) by a uniform distribution with different levels of deviation. Since the focusing efficiency will vary from one sample to the other, we consider the average focusing efficiency and its deviation for 100 samples. Figures S6 and S7 demonstrate the results of focusing efficiency vs wavelength alongside typical nearfield distributions at the operating wavelength of λ=1550 nm corresponding to different levels of deviations in the filling fraction and shell radius of the original design, respectively.

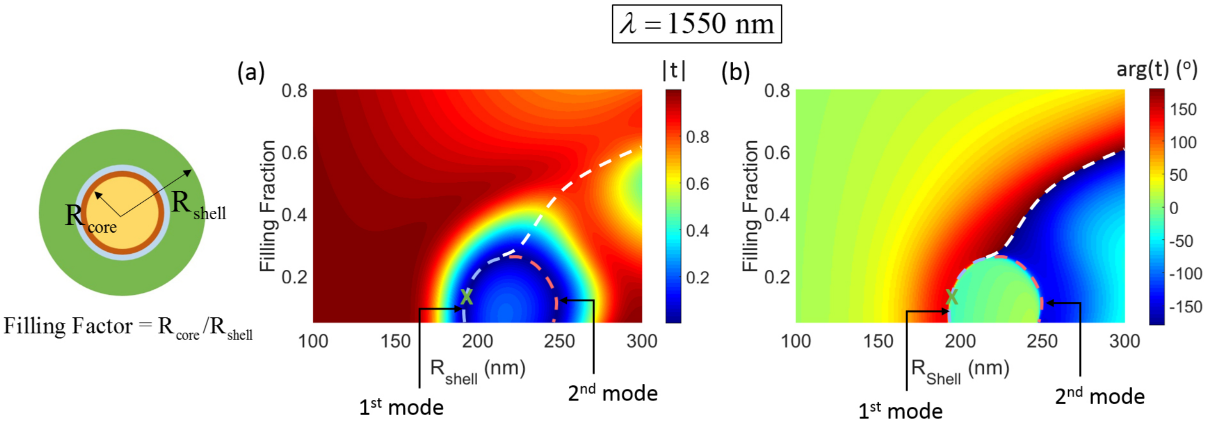

Figure S 4. The transmission (a) amplitude and (b) phase shift as a function of shell radius and filling fraction of the metallic core in the multimaterial nanowire building block. The blue and red dashed lines correspond to the two excited TE resonances, while the white dashed line denotes merging of the resonances.

As it can be observed, the focusing functionality is preserved more or less in all cases while the focusing efficiency degrades significantly as the deviation level in the geometrical parameters increases. The maximum focusing efficiency may also shift into lower and higher wavelengths with respect to the operating wavelength of the original design. Moreover, comparing Figs. S6 and S7, the degradation in the performance is more significant with respect to the deviations in the shell radius. It should be mentioned that even though the structural non-uniformities are inevitable in the fabrication process, the recent advances in drawing and lithography techniques have pushed the resolution of core-shell nanowires into sub-5 nm scale ensuring that a satisfactory performance of the designed metasurface can be achieved in practice. It should be also remarked that a further improvement of performance can be made possible in the future by the emergence of novel tunable materials with lower loss which can relax the restriction on the critical coupling condition and allow for a wider bandwidth of operation.

Alternative Metasurface Designs with Higher Transmission Efficiencies

Here, we propose an alternative design by using asymmetric bi-nanowires as constituent elements of the metasurface which leads to almost $\approx$10 times higher amplitude efficiency comparing to the single nanowire design. This is made possible by the higher transmission level of the proposed structure at the resonant condition. The schematic of the metasurface configuration and its unit-cell with the corresponding geometrical parameters are given in Fig. S8(a). The geometrical parameters of the design are optimized in such a way that the structure exhibits the magnetic resonance at the operating wavelength of 1550 nm at no-bias. Figures S8(b) and (c) compare the performance of this design with the single nanowire counterpart in terms of the transmission phase shift and amplitude, respectively. An almost 10 times higher transmission efficiency could be obtained while still maintaining the large phase shift of over 280 degrees.

Despite the performance improvement by using an alternative geometry for the subwavelength resonators constituting the metasurface, the transmission efficiency is still hindered by being limited to operation at the resonance. This restriction can be relaxed by using improved materials with similar field-modulation effect of ITO but with lower optical loss. Such a material can shift the resonance more significantly through electrical biasing which allows for operating off-resonance or operating at a balanced excitation of multiple resonances similar to Kerker-type scattering at all-dielectric metasurfaces and enables a full-phase coverage with a high transmission efficiency. Here, we consider a hypothetical TCO material with similar parameters to those of ITO except with a damping coefficient of $\Gamma = 1.8$ THz which leads to significant reduction of the material loss. The transport properties in this hypothetical TCO material is approximately modeled by considering a homogeneous charge accumulation layer with a thickness of 2 nm. The background carrier concentration is considered to be 3×10^{20} cm^{-3} while the carrier concentration in the accumulation layer is increased up to 8×10^{20} cm^{-3}. An optimum design is made using this material and the results are shown in Fig. S9. The geometrical parameters of the design are given in Fig. S8(a) and the transmission amplitude and phase shift in terms of carrier concentration of the accumulation layer at the operating wavelength of 1550 nm are plotted in Figs. S8(b) and (c), respectively. As it can be seen, a transmission amplitude higher than 0.6 can be obtained while covering a 2π range for the transmission phase shift. In order to gain more physical insight to the underlying mechanism of the afforded phase shift, the transmission amplitude and phase shift spectra are plotted for different values of the carrier concentration in Figs. S8(d) and (e), respectively. The results clearly demonstrate that the lower loss of the material allows for

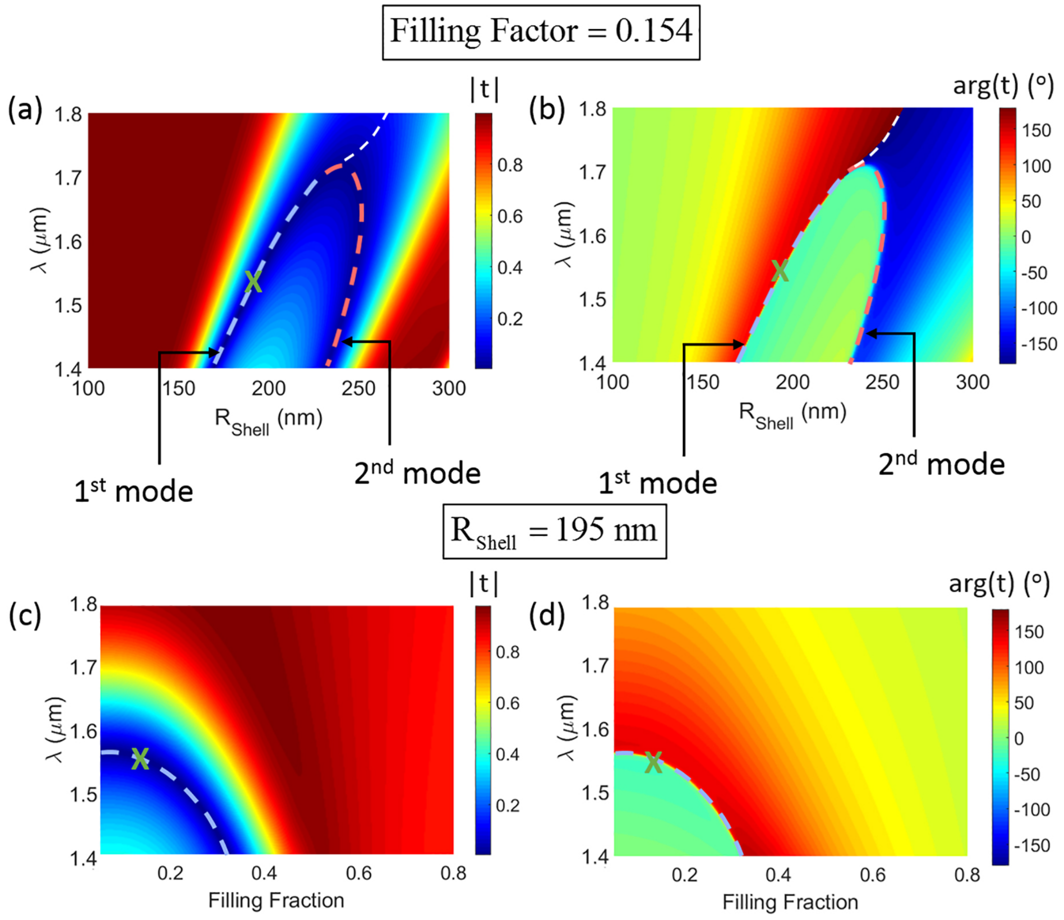

Figure S 5. The spectral transmission (a) amplitude and (b) phase as a function of shell radius for a fixed filling fraction of 0.154. The blue and red dashed lines correspond to the first and second TE resonant modes while the white dashed line denoted merging of the resonances. The parameters of the design are shown by the ”x” marker. (c) and (d) show the same as a function of filling fraction for a fixed shell radius of 195 nm.

more significant changes in the resonance wavelength by coupling to the ENZ region. This enables the possibility of operating between two resonances which can afford a full phase modulation of 2π while maintaining a high transmission amplitude.

It should be remarked that a lossless realization of an ENZ material is not physically possible due to causality, however, with the continuous trend in the discovery of new, high performance plasmonic materials, it is expected that alternative materials with lower loss become available in the future.

I-V Characteristics of the Unit-cells: Power Consumption

The tunability mechanism of ITO-based metamaterials relies on the field-modulation effect which can be achieved by applying a voltage across the ITO layer through an external bias. As it was mentioned in the manuscript, one of the main advantages offered by the field-modulation effect comparing to other tunable mechanisms is its low power consumption by applying external bias. In order to verify this claim, we obtain IV characteristics of the unit-cells proposed in the manuscript. The results are obtained using Cogenda Device Simulator and are shown in Fig. S10. As it can be seen, the leakage currents are extremely small making the power consumption negligible. The cut-off voltage of the two elements differ from each other and both unit-cells are biased below the breakdown voltage.

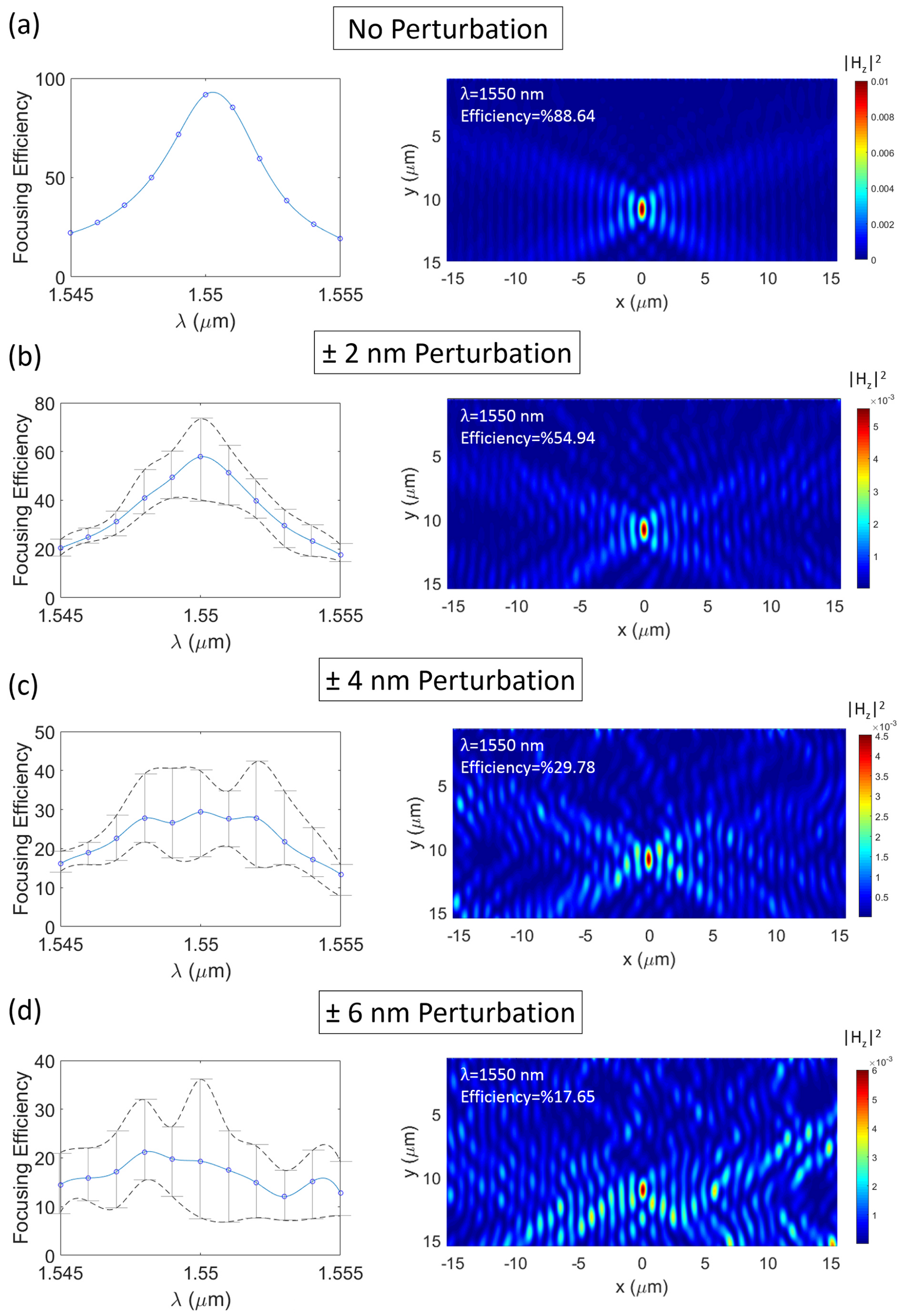

Figure S 6. (a)-(d) demonstrate the average focusing efficiency and its deviations vs wavelength alongside typical nearfield distribution of magnetic field intensity for different levels of random perturbation in the filling fraction of the multimaterial nanowires. The solid blue lines correspond to the average focusing efficiency while the black dashed lines denote the minimum and maximum focusing efficiencies.

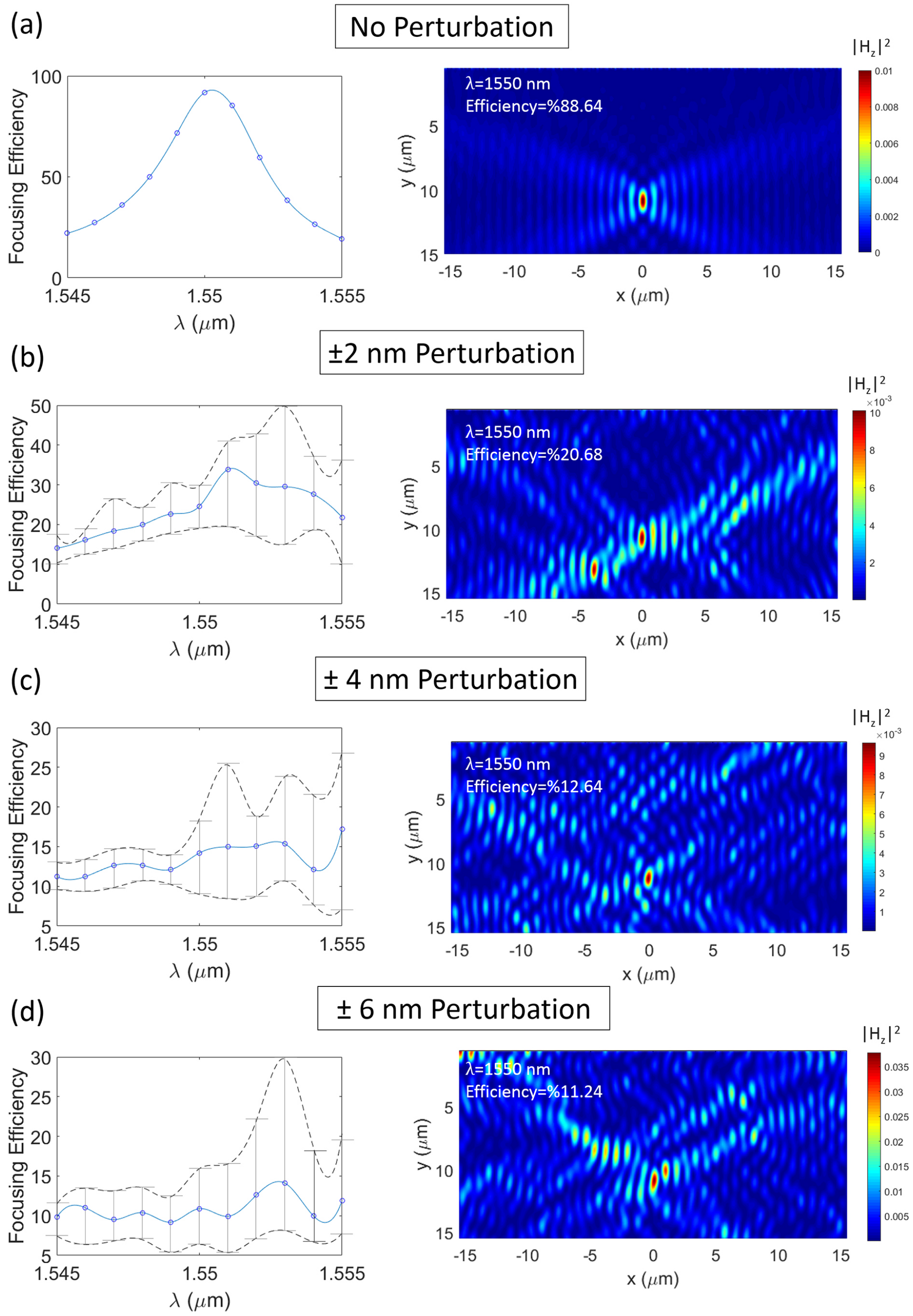

Figure S 7. (a)-(d) demonstrate the average focusing efficiency and its deviations vs wavelength alongside typical nearfield distribution of magnetic field intensity for different levels of random perturbation in the shell radius of the multimaterial nanowires. The solid blue lines correspond to the average focusing efficiency while the black dashed lines denote the minimum and maximum focusing efficiencies.

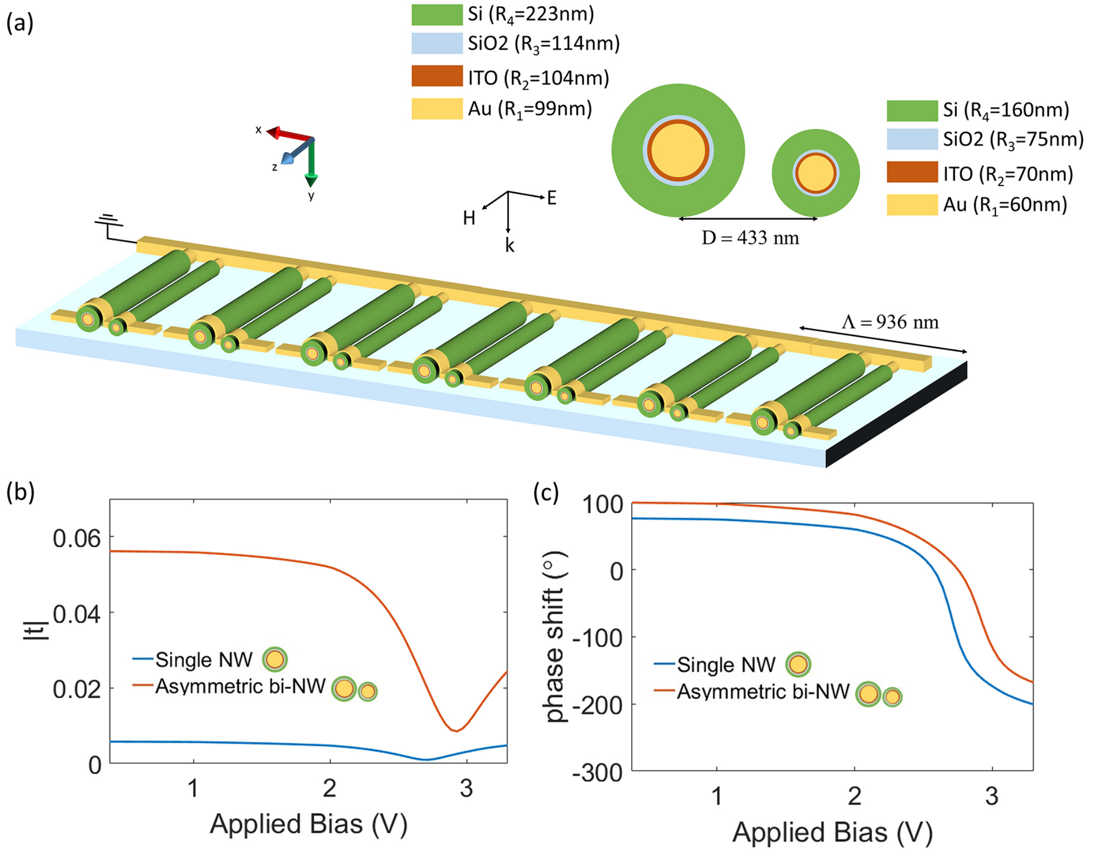

Figure S 8. (a) The geometrical configuration and parameters for a metasurface design consisted of a periodic arrangement of asymmetric bi-nanowires. (b) and (c) compare the transmission amplitude and phase shift as a function of applied bias corresponding to the single nanowire and asymmetric bi-nanowire design.

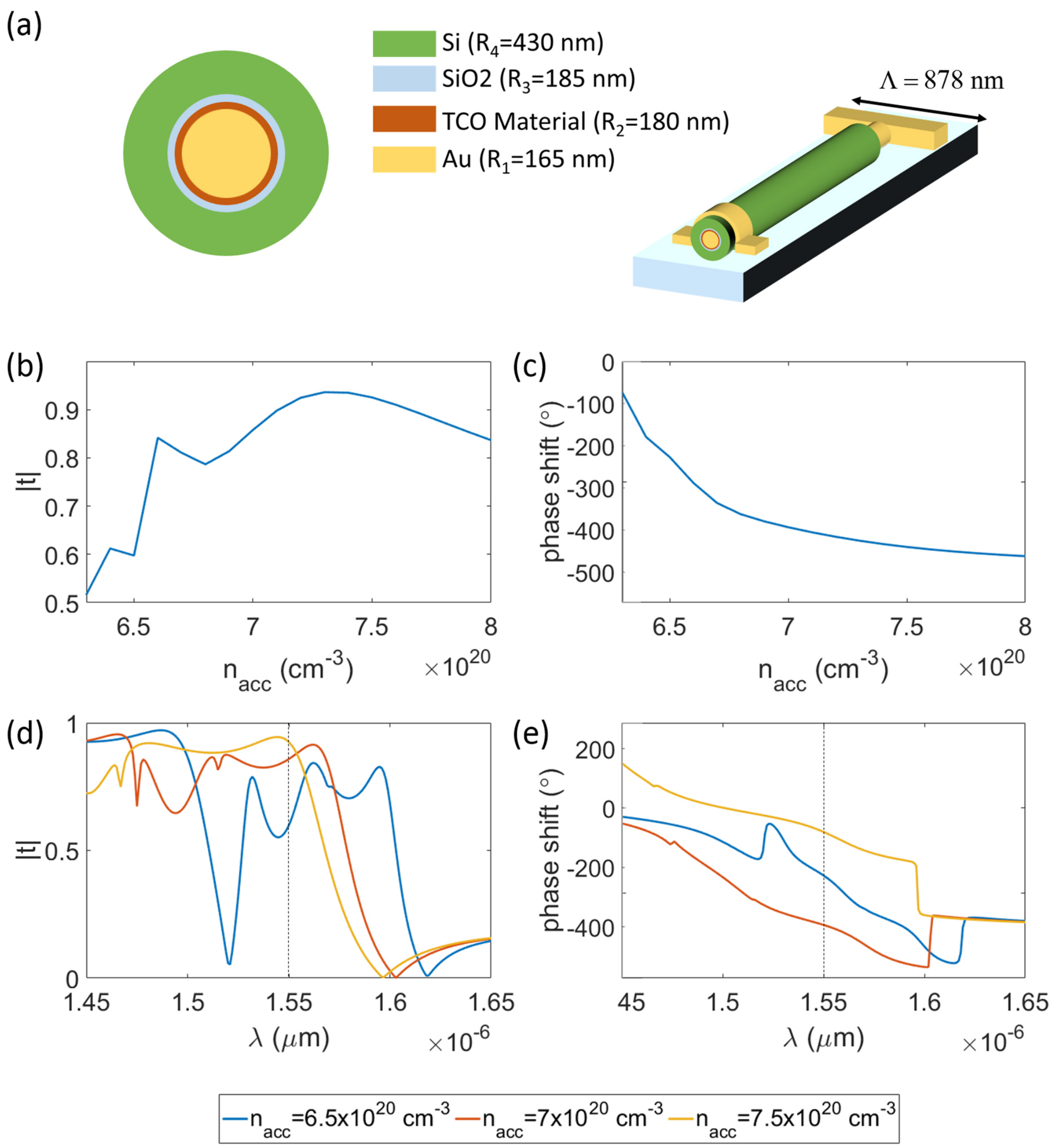

Figure S 9. (a) The geometrical configuration and parameters for a metasurface design consisted of a periodic arrangement of multimaterial nanowires incorporating a hypothetical TCO material with a significantly lower loss comparing to ITO. (b) and (c) compare the transmission amplitude and phase shift as a function of carrier concentration in the charge accumulation layer (The transport properties of the material is modeled by considering a homogeneous charge accumulation layer with a thickness of 2 nm). (d) and (e) demonstrate the transmission amplitude and phase shift spectra for different values of carrier concentration in the accumulation layer.

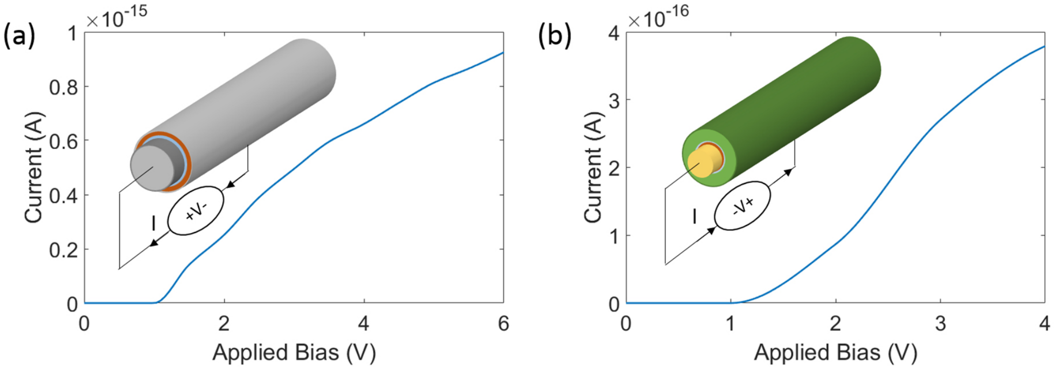

Figure S 10. The IV characteristic of the multimaterial nanowire building blocks used for (a) graded-index metamaterial and (b) metasurface designs.
